# Protocol investigating the clinical utility of an objective measure of activity and attention (QbTest) on diagnostic and treatment decision-making in children and young people with ADHD—‘*A*ssessing *Q*bTest *U*tility in *A*DHD’ (AQUA): a randomised controlled trial

**DOI:** 10.1136/bmjopen-2014-006838

**Published:** 2014-12-01

**Authors:** Charlotte L Hall, Gemma M Walker, Althea Z Valentine, Boliang Guo, Catherine Kaylor-Hughes, Marilyn James, David Daley, Kapil Sayal, Chris Hollis

**Affiliations:** 1 CLAHRC, University of Nottingham, Nottingham, UK; 2 Division of Rehabilitation and Ageing, University of Nottingham, Nottingham, UK; 3 Division of Psychiatry & Applied Psychology, School of Medicine, University of Nottingham, Nottingham, UK; 4 Department of Developmental Psychiatry, University of Nottingham, Queens Medical Centre, Nottingham, UK

**Keywords:** ADHD, Continuous Performance Test (CPT), QbTest, Diagnosis, Treatment

## Abstract

**Introduction:**

The National Institute for Health and Care Excellence (NICE) guidelines for attention deficit/hyperactivity disorder (ADHD) state that young people need to have access to the best evidence-based care to improve outcome. The current ‘gold standard’ ADHD diagnostic assessment combines clinical observation with subjective parent, teacher and self-reports. In routine practice, reports from multiple informants may be unavailable or contradictory, leading to diagnostic uncertainty and delay. The addition of objective tests of attention and activity may help reduce diagnostic uncertainty and delays in initiating treatment leading to improved outcomes. This trial investigates whether providing clinicians with an objective report of levels of attention, impulsivity and activity can lead to an earlier, and more accurate, clinical diagnosis and improved patient outcome.

**Methods and analysis:**

This multisite randomised controlled trial will recruit young people (aged 6–17 years old) who have been referred for an ADHD diagnostic assessment at Child and Adolescent Mental Health Services (CAMHS) and Community Paediatric clinics across England. Routine clinical assessment will be augmented by the QbTest, incorporating a continuous performance test (CPT) and infrared motion tracking of activity. The participant will be randomised into one of two study arms: QbOpen (clinician has immediate access to a QbTest report): QbBlind (report is withheld until the study end). Primary outcomes are time to diagnosis and diagnostic accuracy. Secondary outcomes include clinician's diagnostic confidence and routine clinical outcome measures. Cost-effective analysis will be conducted, alongside a qualitative assessment of the feasibility and acceptability of incorporating QbTest in routine practice.

**Ethics and dissemination:**

The findings from the study will inform commissioners, clinicians and managers about the feasibility, acceptability, clinical utility and cost-effectiveness of incorporating QbTest into routine diagnostic assessment of young people with ADHD. The results will be submitted for publication in peer-reviewed journals. The study has received ethical approval.

**Trial registration number:**

NCT02209116.

**Strengths and limitations of this study**

- A pragmatic diagnostic randomised controlled trial design in standard National Health Service (NHS) settings.
- The focus of the trial is the impact of an objective assessment technology on clinical decision making and patient outcomes.
- There is limited data regarding the clinical utility, cost-effectiveness and acceptability of using the QbTest to aid attention deficit/hyperactivity disorder assessment and treatment.

## Introduction

Attention deficit/hyperactivity disorder (ADHD) is the most common neurodevelopmental disorder and affects 3–5% of children and young people.^1^ The core symptoms of ADHD include poor attention, hyperactivity and impulsivity. National Institute for Health and Care Excellence (NICE)^1^ guidelines provide a blueprint for the diagnosis and management of ADHD in England and Wales and indicate the need for young people with ADHD to have access to the best evidence-based care in order to fulfil their potential and prevent poor outcome. However, in practice, delivery and quality of care is ad hoc, with little consistency in assessment, diagnosis or management.^2^ ADHD frequently coexists with other neurodevelopmental and psychiatric disorders and is a risk factor for major educational, social and occupational impairment, placing a huge burden on the National Health Service (NHS), social care and criminal justice systems. There has been a rapid growth in diagnosis over the past 30 years with the number of children recognised and treated for ADHD in the UK increasing almost 10-fold from the early 1980s^1^ and spending on medication for ADHD increasing sevenfold between 1998 and 2005.^3^ The cost of initial specialist assessment for ADHD is estimated at £23 million annually in England and Wales^4^ and drug costs for ADHD in England during 2012 was expected to exceed £78 million^3^ while indirect costs to families include parental mental ill health, time off work and loss of earnings are even higher.^5^ Increasing recognition of ADHD as a lifespan condition is placing a new demand on the NHS to provide diagnostic and treatment services for children, adolescents and adults, exposing serious limitations in existing methods of assessment and management.

There is no single test, or biomarker used to diagnose the disorder.^6^ In the absence of any objective measure to identify ADHD, clinical assessment and diagnosis is based on the clinician's integration of various forms of subjective information including direct observation and reports from parents, teachers and young people. This approach is heavily reliant on subjective measures and clinical interpretation, which can lead to lack of reliability and consistency in the diagnosis of ADHD^7^ and furthermore, the process of ‘gold standard’ clinical interviews and data collection from multiple informants is time consuming and often difficult to conduct in real world settings with frequent missing data and inconsistencies between reports leading to and diagnostic uncertainty and delay.

Additionally, while treatments for ADHD are highly efficacious in carefully managed research settings^1^ in standard community care the outcome of treatment may be suboptimal. Aside from delays in initiating treatment caused by diagnostic uncertainty, once on medication, children may not be reviewed sufficiently frequently for clinicians to detect non-response or partial response, or to establish the optimal dose for each child. The US National Institute of Mental Health (NIMH) Multimodal Treatment study of ADHD (MTA) showed that careful medication management can significantly improve outcomes, doubling the normalisation rate from 25% in routine community care to almost 60% when using a strategy of careful dose titration and frequent monitoring of outcome.^8^ The NICE^1^ ADHD guidelines recommends that during the titration phase, symptoms should be closely monitored using rating scales. However, audit data within the East Midlands showed that community care for ADHD falls well below the standards for titration and monitoring set out in the MTA and NICE guidelines (CLAHRC-NDL, 2013, unpublished audit). A further consequence of suboptimal treatment response in routine care is poor medication adherence. In the UK, 50% of patients have stopped ADHD medication after 18 months and 80% after 3 years.^9^

### Objective assessment measures in ADHD

One approach to improving assessment and outcomes in routine care is to add objective laboratory measures of activity and attention for diagnostic assessment and treatment optimisation.^5^ Objective measures have the potential to augment and streamline current practice in order to shorten assessment time, increase diagnostic accuracy, reduce delays in treatment and optimise treatment response.

### Continuous performance test

A continuous performance test (CPT) is a neuropsychological test that measures the individual's capacity to sustain attention (vigilance) and inhibit inappropriate responses (impulsivity), which can be used alongside clinical evaluation to inform the diagnostic process.^10^ Typically, a CPT is a computer-based programme which involves rapid presentation of visual or auditory stimuli. Participants are asked to respond when a given target occurs but remain passive to non-targets. A standard CPT typically records the child's omission errors (responding when the target is present), commission errors (responding when the target is not present), number of correct responses, reaction time and variability in reaction time. There are several well validated commercially available CPT tests such as the TOVA (Test of Variables of Attention^11^), IVA (Integrated Visual and Auditory^12^), ACPT (Auditory CPT^13^) and the Conner's CPT.^14^ These tests are one of the most popular clinic-based measures to assess sustained attention in children;^15^ with several studies and a meta-analyses showing that children with ADHD perform worse on these tasks than children without ADHD.^16^ CPTs have also been shown to be a sensitive measure of medication effects.^17^ Several studies have noted improvement in CPT scores in children with ADHD on stimulant medication.^15^ ^18–20^ However, little research has compared CPT scores with more subjective measurements of ADHD^7^ and in their recent review Ogundele *et al*^7^ recommended further research in the use of CPTs compared to standard practice to determine cost-effectiveness of these tasks.

A significant limitation of the CPT for the assessment of ADHD is that it does not measure the patients’ activity levels, which is a core symptom domain of ADHD. Approaches to the objective measurement of activity in ADHD have included wrist-worn actigraphy devices and infra-red motion capture.

### QbTest

QbTest (Qbtech Ltd) has been developed to combine a CPT to measure attention and impulsivity with infra-red motion capture of head movement during the CPT to measure activity.^21^ The QbTest CPT requires participants to respond to an infrequently presented stimulus (by pressing a button) but ignore all others. Physical activity is measured during the course of the CPT via an infra-red camera that tracks the path of a reflector attached to the participants head (central midpoint). These elements of the test provide information on each of the three symptom domains of ADHD and provide summary scores for each individual based on deviation from a normative data set based on age group and gender. There are two versions of the task for children and young people; the task for 6–11-year olds is 15 min duration and the task for 12–17-year olds is 20 min duration. The QbTest result is complemented by a clinical evaluation and behavioural observation of events that may affect test performance. The QbTest is not a stand-alone diagnostic tool, but has been approved by the US Food and Drug Administration (FDA; Ref: K133382) to supplement standard clinical assessment and treatment follow-up by reducing reliance on measures such as subjective observer reports (which can be biased, incomplete or missing) and augment clinical decision-making.

The data available about the QbTest has shown favourable psychometric characteristics with child participants. For example, Qbtech report data that establishes 85% sensitivity of classification and 92% specificity, and good test–retest scores, which is not influenced by experience with computers (F Ulberstad, 2012, unpublished data). Reh *et al*^22^ investigated the factor structure and concurrent and discriminant validity of QbTest and found the hyperactivity factor correlated with teacher ratings of hyperactive behaviour, providing evidence for the utility of including this additional measure of activity in a CPT. In addition, Reh *et al*^23^ found the hyperactivity factor could identify intermediate levels of impairment in ADHD siblings, suggesting this factor maybe particularly sensitivity as an intermediate phenotype for ADHD. Their findings also provide initial evidence for the concurrent validity of the three factors (attention, impulsivity and activity), although the authors highlight the need for further research to investigate validity. Wehmeier *et al*^24^ found QbTest to be a valid measure of treatment outcome and highly correlated with blinded observer ratings of behaviour in placebo-controlled randomised controlled trial (RCTs). QbTest is effective in evaluating ADHD medication effects in children^25^ ^26^ and can identify early non-responders.^15^ One clinical study found QbTest improved clinical accuracy by reducing the risk of unidentified ADHD when patients were re-evaluated 1 year after their initial assessment^27^ and another indicated the ability for QbTest to differentiate ADHD from normative controls.^28^ Initial audit data (K Selby, 2013, unpublished data) suggest that implementation of QbTest in routine ADHD clinics can reduce the time to diagnosis by 30%. This equates to a reduction from an average of three to two out-patient appointments per patient in order to either confirm or exclude a diagnosis of ADHD. These findings indicate potential for QbTest to support the diagnostic assessment and management of ADHD within routine clinical practice; however, there has been no RCT to investigate the added clinical value (clinical utility) and economic cost-effectiveness of adding QbTest to standard ADHD care pathways within the NHS.

The primary aim of the *A*ssessing *Q*bTest *U*tility in *A*DHD-Trial (AQUA-Trial) is to determine whether using QbTest in routine NHS settings can accelerate diagnosis without compromising diagnostic accuracy. Second, the study aims to examine whether QbTest improves the medication titration process by increasing the proportion of patients normalised after 6 months postbaseline assessment and improves patient outcome. The study will also use qualitative methods to explore the barriers, drivers and facilitators to the adoption of the QbTest in routine practice. The cost-effectiveness of implementing the QbTest in practice will also be investigated. The findings will indicate whether establishing QbTest as part of standard practice in ADHD assessment and management is clinically useful, financially viable and acceptable for clinicians and patients.

## Methods and analysis

### Trial design

This study is a parallel group single-blind multicentre RCT, exploring feasibility and acceptability, using quantitative and qualitative and health economic evaluations. The study consists of two arms:

1. QbOpen (QbO): In this arm of the trial participants will complete the QbTest and clinicians, participants and their families will have immediate access to a QbTest report.
2. QbBlind (QbB): In this arm of the trial participants will complete the QbTest but the QbTest report will be withheld from the clinician, participant and patient's family until the last outcome measure is completed at 6 months. All participants will receive the same intervention. Specifically, this will be assessment as usual plus a QbTest as part of the diagnostic assessment.

The patients usual care team will be responsible for conducting the QbTest in clinic appointments. The QbTest will be only be conducted by trained QbTest clinicians.

### Setting

Child and Adolescent Mental Health Services (CAMHS) and Community Paediatric clinics across nine different NHS Trusts in England, including Medway NHS Foundation Trust, Alder Hey Children's NHS Foundation Trust, Nottinghamshire Healthcare NHS Trust, Leicestershire Partnership NHS Trust, Sussex Partnership NHS Foundation Trust, United Lincolnshire Hospital NHS Trust, Central Manchester University Hospitals NHS Foundation Trust, Bridgewater Community Healthcare NHS Trust, Nottingham University Hospitals NHS Trust. Additional NHS Trusts may be recruited to meet target recruitment figures.

### Recruitment and eligibility

New referrals for a diagnostic evaluation for suspected ADHD will be invited to participate in the research based on the following criteria:

*Inclusion criteria*

- Age 6–17 years old (at the time of consent);
- Referred to CAMHS or Community Paediatrics for an ADHD diagnostic assessment;
- Capable of providing written informed consent (over 16 years old);
- Parental consent (under 16 years old).

*Exclusion criteria*

- Severe learning disability (to be assessed by clinical judgment);
- Non-fluent English speaking;
- Previous or current confirmed diagnosis of ADHD;
- Currently receiving ADHD medication.

Written information about the trial will be sent to families through the clinic administrators prior to their first appointment. Clinic invitations will be updated and recorded on a password protected database on a weekly basis. Parents and young people who wish to participate will be asked to complete and return a consent form before this appointment. Alternatively, participants will be consented into the study by the clinical team, clinical studies officer or a member of the research team at their first appointment. Each site will be informed of the monthly recruitment target required in order to meet the study sample size and updated on their monthly progress.

### Trial phases

**Figure 1:**
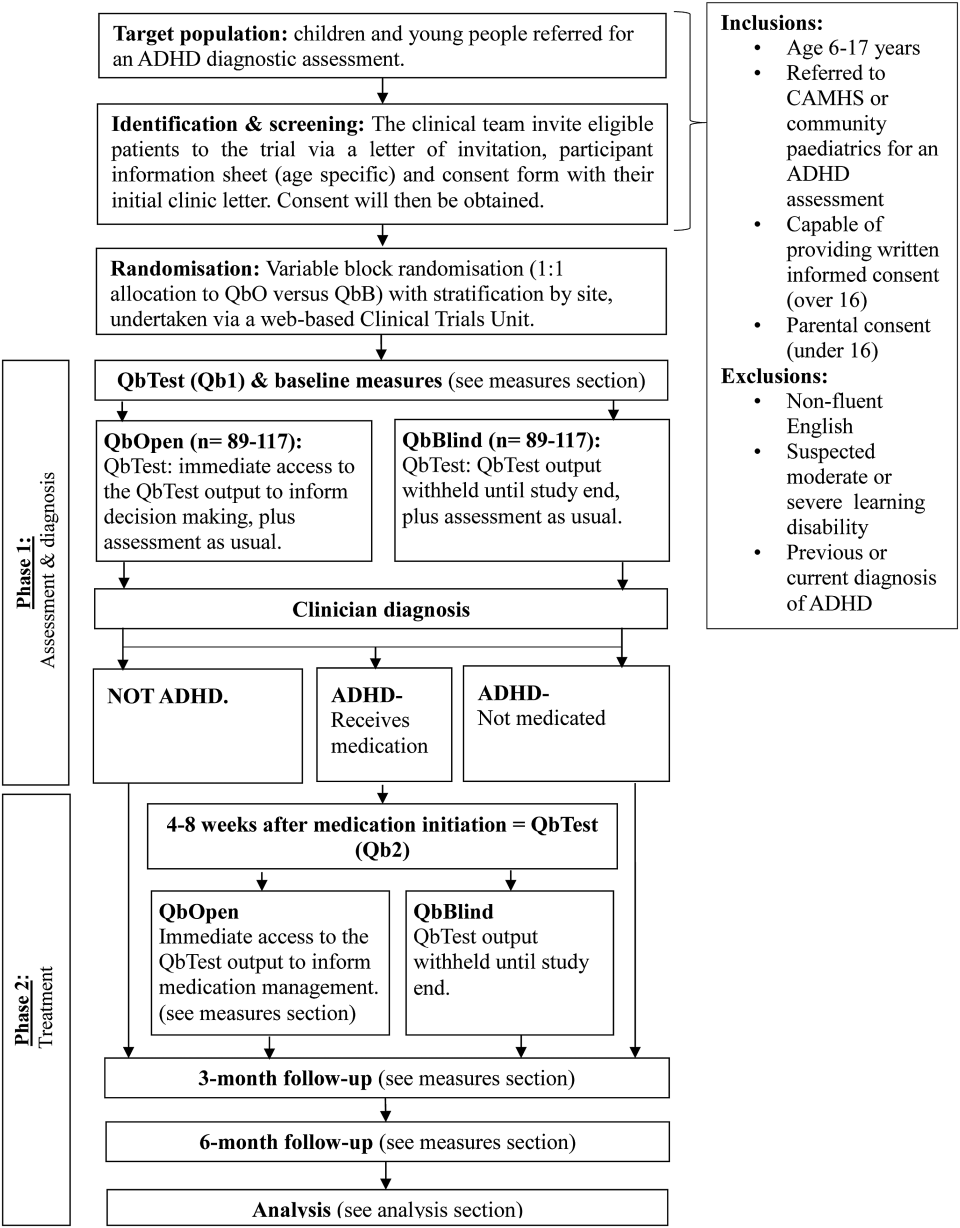
Study flow chart. ADHD, attention deficit/hyperactivity disorder; CAMHS, Child and Adolescent Mental Health Services.

There are two phases to this study (figure 1).

Phase 1, Assessment: The first phase investigates the use of QbTest as a tool to aid diagnosis. Participants will undergo ADHD ‘assessment as usual’, which varies between clinics, clinicians and cases, but will typically involve an interview with the parent/young person and collection of questionnaires from the parent/young person and teacher. While attending the clinic, the participant will be asked to complete the QbTest at some point during the diagnostic process. Participants will also be asked to complete baseline outcome measures (see measures section).

Phase 2, Treatment: Patients who receive a clinic diagnosis of ADHD and are allocated by clinicians to receive ADHD medication initiated within 3 months of their baseline assessment will be asked to complete a second QbTest (Qb2) 4–8 weeks after medication initiation. This timeframe was chosen to ensure that all participants can complete their second QbTest before the 6-month follow-up.

All participants will stay in the trial for 6 months and will be asked to complete outcome measures at 3 and 6-month follow-up, regardless of their diagnosis or whether they receive medication. With the aim of promoting participant retention and completion of follow-up measures, participants will be compensated for their time with a £15 high-street voucher if they remain in the trial until 6 months.

### Measures

Blinded members of the research team (CLH, GMW, AZV,) will be fully trained in all trial assessments and responsible for monitoring the distribution, completion and collection of all outcome measures.

#### Primary outcome

The primary outcome is the number of consultations until a confirmed clinical diagnosis is reached, as recorded on a short pro-forma. The pro-forma will be completed by clinicians after each consultation with the young person and/or family and documents information about appointment duration, diagnosis and medication/treatment. The pro-forma can be provided by contacting the corresponding author.

#### Secondary outcomes

The secondary outcomes obtained from the pro-forma are:

- Number of days and duration of visits (in minutes) until a confirmed diagnosis is reached.
- Clinical confidence in diagnostic decision. Clinicians will be required to rate the confidence of their decision on a 7-point Likert scale (Definitely ADHD-Definitely not ADHD).
- Stability in diagnosis. Clinicians will be required to re-rate their diagnostic decision and confidence at 6 months.

#### Other measures

- Development and Well-being Assessment (DAWBA^29^): The DAWBA is a semistructured, investigator-based diagnostic interview for child mental health problems, including ADHD, which includes the (Strengths and Difficulties Questionnaire; SDQ^30^) as an initial screen. The parent and teacher DAWBA will be completed to compare the accuracy of clinic diagnosis (in QbO and QbB arms of the trial) to that of an independent clinical consensus diagnosis made using the DAWBA. Two experienced clinicians, blind to allocation, will review the DAWBA and arrive at a clinical consensus diagnosis.
- QbTest scores: Q-scores for attention, impulsivity and activity will be compared with DAWBA ADHD diagnosis to obtain the best predictive model based on QbTest scores that discriminates between ADHD ‘positive’ and ADHD ‘negative’ gold standard DAWBA diagnosis.
- Side-effects scale:^31^ Side-effects scale will be completed as a control check in medicated participants to ensure greater speed to diagnosis/medication normalisation is not off-set by greater side effects.
- SNAP-IV:^32^ The proportion of patients achieving symptom normalisation assessed via the SNAP-IV. If the young person receives a QbTest on medication (Qb2), the timing on the 3-month SNAP-IV will be moved to coincide with Qb2 to provide a direct comparison of subjective (SNAP-IV) and objective (QbTest) measures. The SNAP-IV is a rating scale designed to assess ADHD symptoms.
- SDQ:^30^ The SDQ is a brief behavioural screening questionnaire which can be used as part of a clinical assessment.
- C-GAS (Children's Global Assessment Scale^33^): Clinician opinion of patient outcome will be assessed via the C-GAS. The C-GAS is a 0–100 scale which that integrates psychological, social and academic functioning in children.
- EQ-5D-Y (EuroQol Five Dimensions Heath Questionnaire-Youth^34^): Child health-related quality of life will be assessed using the EQ-5D-Y.
- A resource collection profile tool will be used. It will encompass elements of a CSRI (Client Service Receipt Inventory^35^) often used in mental health studies but will be a specifically designed economic collection pro-forma for the purpose of this study. It will collect demographic details as well as information on all the services used by the child and family borne costs to be estimated. Indirect costs such as time lost from work incurred by the child's parents or carers will further be recorded. This measure will enable a societal wide perspective for a cost-effectiveness analysis of the QbTest. The DAWBA^29^ QbTest^22^ SDQ^30^ Side-effects scale,^31^ SNAP-IV^32^ C-GAS^33^ EQ-5D-Y^34^ and CSRI^35^ all have established reliability, validity and history of use in clinical and research settings.

#### Feasibility and acceptability

QbTest opinion questionnaire and interview: Clinician and patient opinion of the QbTest will be assessed via a questionnaire, developed by CLH and currently used to assess QbTest opinion in on-going studies at the Queens Medical Centre, Nottingham. This will provide information on the acceptability of QbTest in routine NHS settings. A subsample (n=20) of families and clinicians will be invited to participate in qualitative interviews to further explore acceptability and feasibility of the QbTest. The subsample will be chosen at random from each participating site, using a random number generator.

Table 1 displays a summary of measures, the informant and the time point of completion. All measures will have a 1-month window for completion, with the exception of the clinic pro-forma which must be completed during or just after the clinic appointment and the QbTest which must form part of the diagnostic or medication assessment. The collected outcome measures for participants who drop out the trial before 6 months will be included in analysis, no further outcome measures will be collected from these participants after they terminate their participation.

**Table 1:** Synoptic table of study measures

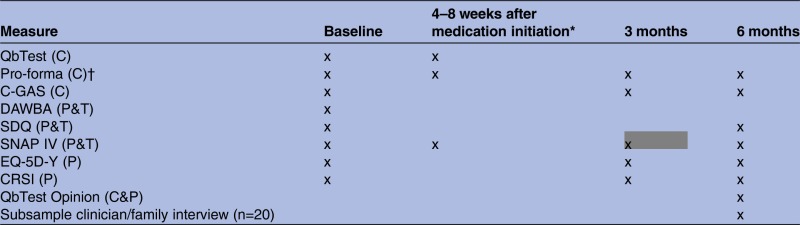

*If ADHD diagnosis record and medicated within 3 months of baseline assessment (in place of corresponding 3 month assessment in grey).

†Completed at every appointment.

C, clinician completed; P, parent/carer completed; T, teacher completed.

### Sample size and justification

The sample size calculation was based on an audit study data from the Department of Community Pediatrics at the Medway NHS Trust (K Selby, 2013, unpublished data). Calculations based on this audit study data showed that the mean number of visits needed to achieve an ADHD diagnosis before introduction of the QbTest (control rate) for children aged 6–14 year olds was 2.94 visits and following the introduction of QbTest a diagnosis was reached in a mean of 2.18 visits. Following consultation with stakeholders, it was agreed that this difference (2.94–2.18) represented the minimum clinically important difference, with any smaller difference in mean clinic visits being of debatable value. Therefore, 71 patients in each study group will be required to detect a mean count difference of the above magnitude with 80% power at two tailed 0.05 significance level^36^ ^37^ assuming the number of visits follows a Poisson distribution.

Given the evidence that the intraclass correlation coefficients of mental health measures across General Practitioner (GP) centres is extremely low,^38–40^ and results from the Medway audit data indicate that the number of visits needed to achieve an ADHD diagnosis was homogeneous across centres, we will assume that centre effects will not influence the sample size calculation for this study. After taking into account a 20% attrition rate, the final total sample size will be 178. The same calculation performed with 90% power would require a total sample of 234 participants. We aim to recruit 178 participants as a minimum and 234 participants as a maximum. Software Stata V.13 was used for power analysis.

### Randomisation and blinding

Once consent has been obtained from participants, their information will be entered onto a web-based randomisation system (set up by University of Nottingham Clinical Trials Unit; CTU). The arm to which a participant is assigned will be determined by a computer generated pseudo-random code using random permuted blocks of varying size, created by the Nottingham CTU in accordance with their standard operating procedure and held on a secure server. Participants will be allocated with equal probability to each arm (QbO and QbB) with stratification by site. All participants will undergo the same research measures, including the QbTest. It is the time at which the report is made available to the clinician and patient that is randomised (immediately vs 6 months later). Outcome assessors for all measures will be blind to which arm the participant is in. There are no anticipated events.

In which participant unblinding would be necessary. There is an allocated unblinded research team member (CKH) to provide control checks where required.

### Data analysis plan

The analysis will be conducted on an intention-to-treat (ITT) basis. Exploratory analysis will be conducted first for outcome and patient background variables; descriptive statistics of each variable will be presented separately for each group at each follow-up point, with means and SD for normally distributed variables, medians (IQR) for skewed variables and frequency (percentage) for categorical variables. Missing values will be checked and reported. Multiple imputation will be used to hand missing values, based on a multilevel modelling approach.

To compare the number of visits needed to achieve an ADHD diagnosis (either confirmed or excluded) between groups, Poisson regression with binary group status as the explanatory variable will be implemented. To compare clinician's confidence in their diagnostic decisions, multilevel modelling with patient as a level 2 unit will be used to take into account the non-independence within patient data due to repeated measures.^41^

κ Statistics will be used to reflect the stability of diagnosis between first confirmed diagnosis and diagnosis rerated at 6-month follow-up time. κ Statistics will be reported for each group and the stability of diagnosis will be compared between arms using logistic regression. The same analysis approach will be implemented to explore the stability of diagnosis confidence between time of first confirmed diagnosis and 6-month follow-up. To assess the diagnosis accuracy, the sensitivity, specificity, likelihood ratio (LR) ve+, LR ve−, positive predictive value (PPV) and negative predictive value (NPV) will be reported for each group and the test performance will be compared between QbO and QbB arms.^42^ ^43^ Receiver operating characteristic curve analyses will be used to obtain the best predictive model based on QbTest scores that discriminates between ADHD ‘positive’ and ADHD ‘negative’ gold standard DAWBA diagnoses.

For treatment related outcomes (phase 2) outcome measures such as SNAP-IV, side effects scale, SDQ and C-GAS scores, multilevel modelling with patient as a level 2 unit will be again applied to quantify the difference between QbO and QbB arms.

For time to event variables such as time to diagnosis (in days), survival analysis using log-rank test will be performed for group comparison and Kaplan-Meier survival curves will be displayed for each group. Logistic regression will be used to compare the proportion of normalisation between two groups at 6-month follow-up time. For all regression modelling to explore the difference between arms, group status will be included as explanatory variables. Data transformation would be needed for skewed outcome variables.

### Health economic evaluation

Economic evaluation will be completed primarily from a health service perspective but in addition from a societal perspective. A cost-effectiveness and cost utility analysis of the treatment options will be conducted. This will include incremental cost-effectiveness ratio of QbTest versus usual care; and present cost-effectiveness acceptability curves (CEACs) for the diagnostic/treatment options. CEACs enable a probabilistic visual interpretation of the health economic analysis that can be used by decision-makers to assist in their choice of health service delivery.

### Implementation

To assess feasibility and acceptability we shall look at scores on the QbTest feedback questionnaires. High scores will be taken to indicate high acceptability and feasibility. Mean scores for individual items on each questionnaire will be assessed to determine which aspects of QbTest are perceived negatively or positively by clinicians and service users. Data from clinicians and patients who participate in interviews will be thematically analysed according to the principles of Braun and Clarke^44^ to assess themes on the acceptability of QbTest, including patients’ opinion on reduced length or number of clinic visits.

### Data monitoring

No interim analysis or analyses for safety or efficacy are planned. Access to data will be restricted to trial team members and associated regulatory authorities as indicated in the sponsor agreement between sites and individual participant information sheets. The chief investigator (CH) shall oversee study management, with oversight from the rest of the research team. A sample (10% of the data) will be checked on a regular basis for verification of all entries made. Where corrections are required these will carry a full audit trail and justification, independent from the research team. There are no anticipated adverse effects of the QbTest, all adverse events will be recorded and monitored and the CH will determine seriousness and causality and report the event to the ethics committee.

The trial is overseen by an independent CLAHRC East Midlands Scientific Committee. The members of the committee are drawn externally from outside the institutions of the research team members and the trial sponsor.

## Study limitations

The diagnosis and management of ADHD is inconsistent, as such the ‘assessment as usual’ practice will vary across sites. In order to document this difference each site completed a questionnaire prior to their participation in the trial detailing their ‘assessment as usual’ procedure. Furthermore, basic descriptions of ‘assessment as usual’ will be recorded in the pro-forma (such as number and length of appointments, decision-making and medication). Given this is a pragmatic trial conducted in real-world settings we are interested in the impact of adding QbTest feedback to ‘assessment as usual’—without changing other aspects of practice. In order to minimise the trial results being influenced by practice in any one site, we are recruiting participants across multiple sites in different regions of the country and include both CAMHS and community paediatrics. In our design, we have attempted to control for variations between sites by stratification of randomisation by site.

## Ethics and dissemination

Patient recruitment has started at these sites and additional R&D approvals at other sites are in progress. The study is sponsored by the University Of Nottingham; neither the sponsor nor the funders will be involved in the analysis of study data or report writing. QbTech will provide QbTest reports to the study team, which will be analysed by BG, from the University Of Nottingham. Only the research team will have access to the study data, data generated from the trial will be available for inspection by the ethics and R&D committees on request. Changes to the protocol will be communicated to the ethics committee by the lead research fellow (CLH). The process for obtaining participant informed consent or assent and parent/guardian informed consent will be in accordance with the ethical guidance, and Good Clinical Practice. The investigator or their nominee and the participant or other legally authorised representative (such as the child's parent) shall sign and date the informed consent forms (see online supplementary appendix A and B) before the person can participate in the study. Written consent will be required from young people aged 16 years and above and their parents. If the young person is under 16 years of age, parental consent will be required, with the young person's written or verbal assent. Individual participant medical information obtained as a result of this study are considered confidential and disclosure to third parties is prohibited unless warranted by an adverse event. Participant confidentiality will be further ensured by utilising identification code numbers to correspond to treatment data in the computer files. No post-trial care is required.

The primary aim of this study is to determine whether using QbTest in routine NHS settings can accelerate time to correct diagnosis, with a secondary aim of examining whether the QbTest can improve patient outcome. Currently, there are few trials conducted in routine NHS settings with the aim of improving the ADHD care pathway, despite evidence to suggest suboptimal care standards and rising socioeconomic burdens. The findings of this study will help to demonstrate whether the QbTest is clinically useful and financially viable in standard care. The findings of the trial will be submitted for publication in appropriate journals regardless of outcome (in accordance with the recommendations of CONSORT) and to members of the public.

## Supplementary Material

Author's manuscript

Reviewer comments
