# Supplementary material for: Protocol investigating the clinical utility of an objective measure of activity and attention (QbTest) on diagnostic and treatment decision-making in children and young people with ADHD—‘Assessing QbTest Utility in ADHD’ (AQUA): a randomised controlled trial
Source: BMJ Open. 2014 Dec 1;4(12):e006838. doi: 10.1136/bmjopen-2014-006838 (PMC4256543; doi:10.1136/bmjopen-2014-006838)
Supplement: Author's manuscript [file bmjopen-2014-006838.draft_revisions.pdf]

BMJ Open

Protocol investigating the clinical utility of an objective measure of activity and attention (QbTest) on diagnostic and treatment decision making in children and young people with ADHD: A randomised controlled trial

Journal:	*BMJ Open*
Manuscript ID:	bmjopen-2014-006838
Article Type:	Protocol
Date Submitted by the Author:	06-Oct-2014
Complete List of Authors:	Hall, Charlotte; Institute of Mental Health, NIHR CLAHRC-East Midlands, Psychiatry<br>Walker, Gemma; Institute of Mental Health, Psychiatry<br>valentine, althea; University of Nottingham,<br>Guo, Boliang; Institute of Mental Health, Psychiatry<br>Kaylor-Hughes, Catherine; Institute of Mental Health, Psychiatry<br>james, marilyn; University of Nottingham, ; Institute of Mental Health, Psychiatry<br>daley, david; Institute of Mental Health, Psychiatry<br>Sayal, Kapil; University of Nottingham, ; Institute of Mental Health, Psychiatry<br>Hollis, Chris; Institute of Mental Health, Psychiatry; University of Nottingham, Division of Psychiatry
**Primary Subject Heading**:	Mental health
Secondary Subject Heading:	Health economics, Paediatrics
Keywords:	ADHD, Continuous Performance Test (CPT), QbTest, Diagnosis, Treatment

SCHOLARONE™ Manuscripts

Protocol investigating the clinical utility of an objective measure of activity and attention (QbTest) on diagnostic and treatment decision making in children and young people with ADHD: A randomised controlled trial

Authors

[1]Charlotte L. Hall, Research Fellow, CLAHRC, University of Nottingham, Nottingham, UK

[2]Gemma M. Walker, Research Assistant, CLAHRC, University of Nottingham, Nottingham, UK

[3]Althea Z. Valentine, Researcher, CLAHRC, University of Nottingham, Nottingham, UK

[4]Boliang Guo, Medical Statistician, CLAHRC, University of Nottingham, Nottingham, UK

[5]Catherine Kaylor-Hughes, Clinical Trials Manager, CLAHRC, University of Nottingham, Nottingham, UK

[6]Marilyn James, Professor of Health Economics, Division of Rehabilitation and Ageing, University of Nottingham, Nottingham, UK

[7]David Daley, Professor of Psychological Intervention and Behaviour Change, Division of Psychiatry & Applied Psychology, School of Medicine, University of Nottingham, Nottingham, UK

[8]Kapil Sayal, Professor of Child & Adolescent Psychiatry, Developmental Psychiatry, University of Nottingham, Queens Medical Centre, Nottingham, UK

[9]Chris Hollis, Professor of Child & Adolescent Psychiatry, Developmental Psychiatry, University of Nottingham, Queens Medical Centre, Nottingham, UK

Corresponding Author

[9]Chris Hollis,

Institute of Mental Health

University of Nottingham

Triumph Road

Nottingham, NG7 2TU

Chris.hollis@nottingham.ac.uk

0115 8230258

Key words: ADHD, Continuous Performance Test (CPT), QbTest, diagnosis, treatment

Word count: 4321 (excluding title page, abstract, summary, references and figures)

For peer review only

ABSTRACT

Introduction

The National Institute for Health and Care Excellence (NICE) guidelines for attention deficit/hyperactivity disorder (ADHD) state that young people need to have access to the best evidence-based care to improve outcome. The current 'gold standard' ADHD diagnostic assessment combines clinical observation with subjective parent, teacher and self-reports. In routine practice, reports from multiple informants may be unavailable or contradictory, leading to diagnostic uncertainty and delay. The addition of objective tests of attention and activity may help reduce diagnostic uncertainty and delays in initiating treatment leading to improved outcomes. This trial aims to investigate whether providing clinicians with an objective report of levels of attention, impulsivity and activity can lead to an earlier, and more accurate, clinical diagnosis and improved patient outcome.

Methods and analysis

This multi-site randomised controlled trial (RCT) will recruit young people (aged 6-17-years old) who have been referred for an ADHD diagnostic assessment at Child and Adolescent Mental Health Services (CAMHS) and Community Paediatric clinics across England. Routine clinical assessment will be augmented by the QbTest, incorporating a computerized continuous performance test (CPT) and infrared motion tracking of activity. The participant will be randomised into one of two study arms: QbOpen (clinician has immediate access to a QbTest report): QbBlind (report is withheld until the study end). Primary outcomes are time to diagnosis and diagnostic accuracy. Secondary outcomes include clinician's diagnostic confidence and routine clinical outcome measures. Cost-effective analysis will be conducted, alongside a qualitative assessment of the feasibility and acceptability of incorporating QbTest in routine practice.

Ethics and dissemination

The findings from the study will inform commissioners, clinicians and managers about the feasibility, acceptability, clinical utility and cost effectiveness of incorporating QbTest into routine diagnostic assessment of young people with ADHD. The study has received ethical approval from NRES West Midlands – Coventry and Warwick.

For peer review only - http://bmjopen.bmj.com/site/about/guidelines.xhtml

Trial registration: NCT02209116

ARTICLE SUMMARY

Article focus

- Multi-site pragmatic randomised controlled trial (RCT) investigating the clinical utility of adding QbTest to the standard assessment and management of attention deficit/hyperactivity disorder (ADHD) in young people in routine NHS settings.

Key messages

- There has been significant increase in the diagnosis and treatment of ADHD in the past 30 years, placing additional demands on the NHS.
- The current 'gold standard' ADHD diagnostic assessment combines clinical observation with subjective parent, teacher and self-reports. However, in routine NHS practice, informant reports are often unavailable or contradictory, leading to diagnostic uncertainty and delay.
- The addition of objective tests of attention and activity may help reduce diagnostic uncertainty and delays in initiating treatment leading to improved outcomes. This study will inform practice by addressing the clinical utility and feasibility of implementing a standardised continuous performance test combined with infra-red motion tracking of activity (QbTest) as part of the diagnostic and management procedure for ADHD.

Strengths and limitations

- A pragmatic diagnostic RCT design in standard NHS settings.
- The focus of the trial is the impact of an objective assessment technology on clinical decision making and patient outcomes.
- There is limited data regarding the clinical utility, cost-effectiveness and acceptability of using the QbTest to aid ADHD assessment and treatment.

For peer review only

INTRODUCTION

Attention Deficit/Hyperactivity Disorder (ADHD) is the most common neuro-developmental disorder and affects 3-5% of children and young people[1]. The core symptoms of ADHD include poor attention, hyper-activity and impulsivity. National Institute for Health and Care Excellence (NICE)[1] guidelines provide a blueprint for the diagnosis and management of ADHD in England and Wales and indicate the need for young people with ADHD to have access to the best evidence-based care in order to fulfil their potential and prevent poor outcome. However, in practice, delivery and quality of care is ad-hoc, with little consistency in assessment, diagnosis or management[2]. ADHD frequently co-exists with other neurodevelopmental and psychiatric disorders and is a risk factor for major educational, social and occupational impairment, placing a huge burden on the NHS, social care and criminal justice systems. There has been a rapid growth in diagnosis over the last 30 years with the number of children recognised and treated for ADHD in the UK increasing almost 10 fold from the early 1980s[1], and spending on medication for ADHD increasing seven fold between 1998 and 2005[3]. The cost of initial specialist assessment for ADHD is estimated at £23 million annually in England and Wales[4], and drug costs for ADHD in England during 2012 was expected to exceed £78 million[3], while indirect costs to families include parental mental ill health, time off work and loss of earnings are even higher[5]. Increasing recognition of ADHD as a lifespan condition is placing a new demand on the NHS to provide diagnostic and treatment services for children, adolescents and adults, exposing serious limitations in existing methods of assessment and management.

There is no single test, or biomarker used to diagnose the disorder[6]. In the absence of any objective measure to identify ADHD, clinical assessment and diagnosis is based on the clinician's integration of various forms of subjective information including direct observation and reports from parents, teachers and young people. This approach is heavily reliant on subjective measures and clinical interpretation, which can lead to lack of reliability and consistency in the diagnosis of ADHD[7] and furthermore, the process of 'gold standard' clinical interviews and data collection from multiple informants is time consuming and often

For peer review only

difficult to conduct in real world settings with frequent missing data and inconsistencies between reports leading to and diagnostic uncertainty and delay.

Additionally, whilst treatments for ADHD are highly efficacious in carefully managed research settings[1], in standard community care the outcome of treatment may be sub-optimal. Aside from delays in initiating treatment caused by diagnostic uncertainty, once on medication, children may not be reviewed sufficiently frequently for clinicians to detect non- or patial-response, or to establish the optimal dose for each child. The U.S. National Institute of Mental Health (NIMH) Multimodal Treatment study of ADHD (MTA) showed that careful medication management can significantly improve outcomes, doubling the normalisation rate from 25% in routine community care to almost 60% when using a strategy of careful dose titration and frequent monitoring of outcome[8]. The NICE[1] ADHD guidelines recommends that during the titration phase, symptoms should be closely monitored using rating scales. However, audit data within the East Midlands showed that community care for ADHD falls well below the standards for titration and monitoring set out in the MTA and NICE guidelines (CLAHRC-NDL, 2013, unpublished audit). A further consequence of sub-optimal treatment response in routine care is poor medication adherence. In the U.K., 50% of patients have stopped ADHD medication after 18 months and 80% after 3 years[9].

Objective Assessment Measures in ADHD

One approach to improving assessment and outcomes in routine care is to add objective laboratory measures of activity and attention for both diagnostic assessment and treatment optimisation[5]. Objective measures have the potential to augment and streamline current practice in order to shorten assessment time, increase diagnostic accuracy, reduce delays in treatment, and optimise treatment response.

Continuous Performance Test

A Continuous Performance Test (CPT) is a neuropsychological test that measures the individual's capacity to sustain attention (vigilance) and inhibit inappropriate responses (impulsivity), which can be used alongside clinical evaluation to inform the diagnostic process[10]. Typically, a CPT is a computer based programme which involves rapid presentation of visual or auditory stimuli. Participants are asked to respond when a given target occurs but remain passive to non-targets. A standard CPT typically records the child's

For peer review only

omission errors (responding when the target is present), commission errors (responding when the target is not present), number of correct responses, reaction time and variability in reaction time. There are several well validated commercially available CPT tests such as the TOVA (Test of Variables of Attention[11]), IVA (Integrated Visual and Auditory[2]), ACPT (Auditory Continuous Performance Test[3]) and the Conner's CPT[14]. These tests are one of the most popular clinic based measures to assess sustained attention in children[16]; with several studies and a meta-analyses showing that children with ADHD perform worse on these tasks than children without ADHD[16]. CPTs have also been shown to be a sensitive measure of medication effects[17]. Several studies have noted improvement in CPT scores in children with ADHD on stimulant medication[15, 18-20] . However, little research has compared CPT scores with more subjective measurements of ADHD[7] and in their recent review Ogundele et al.[7] recommended further research in the use of CPTs compared to standard practice to determine cost effectiveness of these tasks.

A significant limitation of the CPT for the assessment of ADHD is that it doesn't measure the patients' activity levels, which is a core symptom domain of ADHD. Approaches to the objective measurement of activity in ADHD have included wrist-worn actigraphy devices and infra-red motion capture.

QbTest

QbTest (Qbtech Ltd) has been developed to combine a CPT to measure attention and impulsivity with infra-red motion capture of head movement during the CPT to measure activity [21]. The QbTest CPT requires participants to respond to an infrequently presented stimulus (by pressing a button) but ignore all others. Physical activity is measured during the course of the CPT via an infra-red camera that tracks the path of a reflector attached to the participants head (central midpoint). These elements of the test provide information on each of the three symptom domains of ADHD and provide summary scores for each individual based on deviation from a normative dataset based on age group and gender. There are two versions of the task for children and young people; the task for 6-11-year olds is 15 minutes duration and the task for 12-17-year olds is 20 minutes duration. The QbTest result is complemented by a clinical evaluation and behavioural observation of events that may affect test performance. The QbTest is not a stand-alone diagnostic tool, but has been approved by the U.S. Food and Drug Administration (FDA) to supplement standard clinical assessment

For peer review only - http://bmjopen.bmj.com/site/about/guidelines.xhtml

and treatment follow-up by reducing reliance on measures such as subjective observer reports (which can be biased, incomplete or missing) and augment clinical decision-making.

The research available about the QbTest has shown favourable psychometric characteristics with child participants. For example, Qbtech report data that establishes 85% sensitivity of classification and 92% specificity, and good test-retest scores, which is not influenced by experience with computers (Ulberstad, 2012, unpublished data). Reh et al.[22] investigated the factor structure and concurrent and discriminant validity of QbTest and found the hyperactivity factor correlated with teacher ratings of hyperactive behaviour, providing evidence for the utility of including this additional measure of activity in a CPT. In addition, Reh et al.[23] found the hyperactivity factor could identify intermediate levels of impairment in ADHD siblings, suggesting this factor maybe particularly sensitivity as an intermediate phenotype for ADHD. Their findings also provide initial evidence for the concurrent validity of the three factors (attention, impulsivity, activity), although the authors highlight the need for further research to investigate validity. Wehmeier et al.[24] found QbTest to be a valid measure of treatment outcome and highly correlated with blinded observer ratings of behaviour in placebo-controlled RCTs. QbTest is effective in evaluating ADHD medication effects in children[25 26], and can identify early non-responders[15]. One clinical study found QbTest improved clinical accuracy by reducing the risk of unidentified ADHD when patients were re-evaluated one-year after their initial assessment[27] and another indicated the ability for QbTest to differentiate ADHD from normative controls[28]. Initial audit data (Selby, 2013, unpublished data) suggest that implementation of QbTest in routine ADHD clinics can reduce the time to diagnosis by 30%. This equates to a reduction from an average of three to two out-patient appointments per patient in order to either confirm or exclude a diagnosis of ADHD. These findings indicate potential for QbTest to support the diagnostic assessment and management of ADHD within routine clinical practice; however, there has been no randomised controlled trial (RCT) to investigate the added clinical value (clinical utility) and economic cost effectiveness of adding QbTest to standard ADHD care pathways within the NHS.

The primary aim of the Assessing QbTest Utility in ADHD-trial (AQUA-Trial) is to determine whether using QbTest in routine NHS settings can accelerate diagnosis without compromising diagnostic accuracy. Secondly, the study aims to examine whether QbTest improves the medication titration process by increasing the proportion of patients normalised

For peer review only - http://bmjopen.bmj.com/site/about/guidelines.xhtml

after 6 months post baseline assessment and improves patient outcome. The study will also use qualitative methods to explore the barriers, drivers and facilitators to the adoption of the QbTest in routine practice and assess its cost effectiveness. The findings will indicate whether establishing QbTest as part of standard practice in ADHD assessment and management is clinically useful, financially viable and acceptable for clinicians and patients.

METHODS AND ANALYSIS

Trial Design

This study is a parallel group single-blind multi-center RCT, exploring feasibility and acceptability, using quantitative and qualitative and health economic evaluations. The study consists of two arms:

1) QbOpen (QbO)

In this arm of the trial participants will complete the QbTest and clinicians, participants and their families will have immediate access to a QbTest report.

2) QbBlind: (QbB):

In this arm of the trial participants will complete the QbTest but the QbTest report will be withheld from the clinician, participant and patient's family until the last outcome measure is completed at 6 months. All participants will receive the same intervention. Specifically, this will be assessment as usual plus a QbTest as part of the diagnostic assessment.

The patients usual care team will be responsible for conducting the QbTest in clinic appointments. The QbTest will be only be conducted by trained QbTest clinicians.

Setting

Child and Adolescent Mental Health Services (CAMHS) and Community Paediatric clinics across 9 different NHS Trusts in England, including Medway NHS Foundation Trust, Alder Hey Children's NHS Foundation Trust, Nottinghamshire Healthcare NHS Trust, Leicestershire Partnership NHS Trust, Sussex Partnership NHS Foundation Trust, United Lincolnshire Hospital NHS Trust, Central Manchester University Hospitals NHS Foundation

For peer review only - http://bmjopen.bmj.com/site/about/guidelines.xhtml

Trust, Bridgewater Community Healthcare NHS Trust, Nottingham University Hospitals NHS Trust. Additional NHS Trusts may be recruited to meet target recruitment figures.

Recruitment and eligibility

New referrals for a diagnostic evaluation for suspected ADHD will be invited to participate in the research based on the following criteria:

Inclusion criteria

- Age 6-17-years old (at the time of consent)
- Referred to CAMHS or Community Paediatrics for an ADHD diagnostic assessment
- Capable of providing written informed consent (over 16-years old)
- Parental consent (under 16-years old)

Exclusion criteria

- Severe learning disability (to be assessed by clinical judgment)
- Non-fluent English speaking
- Previous or current confirmed diagnosis of ADHD
- Currently receiving ADHD medication

Written information about the trial will be sent to families through the clinic administrators prior to their first appointment. Clinic invitations will be updated and recorded on a password protected database on a weekly basis. Parents and young people who wish to participate will be asked to complete and return a consent form before this appointment. Alternatively, participants will be consented into the study by the clinical team, Clinical Studies Officer or a member of the research team at their first appointment. Each site will be informed of the monthly recruitment target required in order to meet the study sample size and updated on their monthly progress.

Trial Phases

There are two phases to this study (see Figure 1).

Phase 1, Assessment: The first phase investigates the use of QbTest as a tool to aid diagnosis.

Participants will undergo ADHD 'assessment as usual', which varies between clinics,

For peer review only

clinicians and cases, but will typically involve an interview with the parent/young person and collection of questionnaires from the parent/young person and teacher. Whilst attending the clinic, the participant will be asked to complete the QbTest at some point during the diagnostic process. Participants will also be asked to complete baseline outcome measures (see measures section).

Phase 2, Treatment: Patients who receive a clinic diagnosis of ADHD and are allocated by clinicians to receive ADHD medication initiated within 3 months of their baseline assessment will be asked to complete a second QbTest (Qb2) 4-8 weeks after medication initiation. This timeframe was chosen to ensure that all participants can complete their second QbTest before the 6-month follow-up.

All participants will stay in the trial for 6-months and will be asked to complete outcome measures at 3 and 6-month follow up, regardless of their diagnosis or whether they receive medication. With the aim of promoting participant retention and completion of follow-up measures, participants will be compensated for their time with a £15 high-street voucher if they remain in the trial until 6-months.

Measures

Blinded members of the research team (CLH, GMW, AVZ,) will be fully trained in all trial assessments and responsible for monitoring the distribution, completion and collection of all outcome measures.

Primary Outcome

The primary outcome is the number of consultations until a confirmed clinical diagnosis is reached, as recorded on a short pro-forma. The pro-forma will be completed by clinicians after each consultation with the young person and/or family and documents information about appointment duration, diagnosis and medication/treatment. The pro-forma can be provided by contacting the corresponding author.

Secondary Outcomes

The secondary outcomes obtained from the pro-forma are:

- Number of days and duration of visits (in minutes) until a confirmed diagnosis is reached.

For peer review only

- Clinical confidence in diagnostic decision. Clinicians will be required to rate the confidence of their decision on a 7-point Likert scale (Definitely ADHD-Definitely not ADHD).
- Stability in diagnosis. Clinicians will be required to re-rate their diagnostic decision and confidence at 6-months.

Other measures

- Development and Well-being Assessment (DAWBA[29]): The DAWBA is a semi-structured, investigator-based diagnostic interview for child mental health problems, including ADHD, which includes the (Strengths and Difficulties Questionnaire; SDQ[30]) as an initial screen. The parent and teacher DAWBA will be completed to compare the accuracy of clinic diagnosis (in both QbO and QbB arms of the trial) to that of an independent clinical consensus diagnosis made using the DAWBA. Two experienced clinicians, blind to allocation, will review the DAWBA and arrive at a clinical consensus diagnosis.
- QbTest scores: Q-scores for attention, impulsivity and activity will be compared with DAWBA ADHD diagnosis to obtain the best predictive model based on QbTest scores that discriminates between ADHD 'positive' and ADHD 'negative' gold standard DAWBA diagnosis.
- Side-effects scale[31]: Side-effects scale will be completed as a control check in medicated participants to ensure greater speed to diagnosis/medication normalisation is not off-set by greater side-effects.
- SNAP-IV[32]: The proportion of patients achieving symptom normalisation assessed via the SNAP-IV. If the young person receives a QbTest on medication (Qb2), the timing on the 3-month SNAP-IV will be moved to coincide with Qb2 to provide a direct comparison of subjective (SNAP-IV) and objective (QbTest) measures. The SNAP-IV is a rating scale designed to assess ADHD symptoms.
- SDQ[30]: The SDQ is a brief behavioural screening questionnaire which can be used as part of a clinical assessment.

For peer review only

- C-GAS (Children's Global Assessment Scale[33]): Clinician opinion of patient outcome will be assessed via the C-GAS. The C-GAS is a 0-100 scale which that integrates psychological, social and academic functioning in children.
- EQ-5D-Y (EuroQol Five Dimensions Heath Questionnaire-Youth[34]): Child health-related quality of life will be assessed using the EQ-5D-Y.
- A resource collection profile tool will be used. It will encompass elements of a CSRI (Client Service Receipt Inventory[35]) often used in mental health studies but will be a specifically designed economic collection pro-forma for the purpose of this study. It will collect demographic details as well as information on all the services used by the child and family borne costs to be estimated. Indirect costs such as time lost from work incurred by the child's parents or carers will further be recorded. This measure will enable a societal wide perspective for a cost-effectiveness analysis of the QbTest. The DAWBA[29], QbTest[22], SDQ[30,] Side-effects scale[31], , SNAP-IV[32], C-GAS[33], EQ-5D-Y[34] and CSRI[35] all have established reliability, validity and history of use in clinical and research settings.

Feasibility and Acceptability

QbTest opinion questionnaire and interview: Clinician and patient opinion of the QbTest will be assessed via a questionnaire, developed by CLH and currently used to assess QbTest opinion in on-going studies at the Queens Medical Centre, Nottingham. This will provide information on the acceptability of QbTest in routine NHS settings. A sub-sample (*n* = 20) of families and clinicians will be invited to participate in qualitative interviews to further explore acceptability and feasibility of the QbTest.

Table 1 displays a summary of measures, the informant and the time point of completion. All measures will have a one-month window for completion, with the exception of the clinic pro-forma which must be completed during or just after the clinic appointment and the QbTest which must form part of the diagnostic or medication assessment. The collected outcome measures for participants who drop-out the trial before 6-months will be included in analysis, no further outcome measures will be collected from these participants after they terminate their participation.

For peer review only

Table 1. Synoptic table of study measures

Measure	Baseline	4-8 weeks after medication initiation*	3 months	6 months
QbTest (C)	x	x		
Pro-forma (C)**	x	x	x	x
C-GAS (C)	x		x	x
DAWBA(P&T)	x			
SDQ (P&T)	x			x
SNAP IV (P&T)	x	x	x	x
EQ-5D-Y (P)	x		x	x
CRSI (P)	x		x	x
QbTest Opinion (C&P)				x
Sub-sample clinician/family interview (n=20)				x

Note. C = Clinician completed. P = Parent/carer completed. T = Teacher completed. *if ADHD diagnosis record and medicated within 3 months of baseline assessment (in place of corresponding 3 month assessment in grey) **Completed at every appointment.

Sample size and justification

The sample size calculation was based on an audit study data from the Department of Community Pediatrics at the Medway NHS Trust (Selby, 2013, unpublished data). Calculations based on this audit study data showed that the mean number of visits needed to achieve an ADHD diagnosis before introduction of the QbTest (control rate) for children

For peer review only

aged 6-14-year olds was 2.94 visits and following the introduction of QbTest a diagnosis was reached in a mean of 2.18 visits. Following consultation with stakeholders, it was agreed that this difference (2.94-2.18) represented the minimum clinically important difference (MCID), with any smaller difference in mean clinic visits being of debatable value. Therefore, 71 patients in each study group will be required to detect a mean count difference of the above magnitude with 80% power at two tailed 0.05 significance level [36 37], assuming the count of visit follows a Poisson distribution. Assuming the number of visits follows a Poisson distribution.

Given the evidence that the Intraclass Correlation Coefficients (ICC) of mental health measures across GP centers is extremely low[38-40], and results from the Medway audit data indicate that the number of visits needed to achieve an ADHD diagnosis was homogeneous across centers, we will assume that center effects will not influence the sample size calculation for this study. After taking into account a 20% attrition rate, the final total sample size will be 178. The same calculation performed with 90% power would require a total sample of 234 participants. We aim to recruit 178 participants as a minimum and 234 participants as a maximum. Software Stata 13 was used for power analysis.

Randomisation and blinding

Once consent has been obtained from participants, their information will be entered onto a web-based randomisation system (set up by University of Nottingham Clinical Trials Unit; CTU). The arm to which a participant is assigned will be determined by a computer generated pseudo-random code using random permuted blocks of varying size, created by the Nottingham CTU in accordance with their standard operating procedure and held on a secure server. Participants will be allocated with equal probability to each arm (QbO and QbB) with stratification by site. All participants will undergo the same research measures, including the QbTest. It is the time at which the report is made available to the clinician and patient that is randomised (immediately versus 6-months later). Outcome assessors for all measures will be blind to which arm the participant is in. There are no anticipated events

In which participant unblinding would be necessary. There is an allocated un-blinded research team member (CKH) to provide control checks where required.

Data analysis plan

The analysis will be conducted on an Intention to treat (ITT) basis. Exploratory analysis will be conducted first for both outcome and patient background variables; descriptive statistics of

For peer review only - http://bmjopen.bmj.com/site/about/guidelines.xhtml

each variable will be presented separately for each group at each follow-up point, with means and standard deviations for normally distributed variables, medians (inter-quartile range) for skewed variables and frequency (percentage) for categorical variables. Missing values will be checked and reported. Multiple imputation will be used to hand missing values, based on a multilevel modelling approach.

To compare the number of visits needed to achieve an ADHD diagnosis (either confirmed or excluded) between groups, Poisson regression with binary group status as the explanatory variable will be implemented. To compare clinician's confidence in their diagnostic decisions, multilevel modelling with patient as a level 2 unit will be used to take into account the non-independence within patient data due to repeated measures[41].

Kappa statistics will be used to reflect the stability of diagnosis between first confirmed diagnosis and diagnosis rerated at 6-month follow up time. Kappa statistics will be reported for each group and the stability of diagnosis will be compared between arms using logistic regression. The same analysis approach will be implemented to explore the stability of diagnosis confidence between time of 1st confirmed diagnosis and 6-month follow-up. To assess the diagnosis accuracy, the sensitivity, specificity, likelihood ratio (LR) ve+, LR ve-, positive predictive value (PPV) and negative predictive value (NPV) will be reported for each group and the test performance will be compared between QbO and QbB arms[42, 43]. Receiver Operating Characteristic (ROC) curve analyses will be used to obtain the best predictive model based on QbTest scores that discriminates between ADHD 'positive' and ADHD 'negative' gold standard DAWBA diagnoses.

For treatment related outcomes (phase 2) outcome measures such as SNAP-IV, side effects scale, SDQ and C-GAS scores, multilevel modelling with patient as a level 2 unit will be again applied to quantify the difference between QbO and QbB arms.

For time to event variables such as time to diagnosis (in days), survival analysis using log-rank test will be performed for group comparison and Kaplan-Meier survival curves will be displayed for each group. Logistic regression will be used to compare the proportion of normalisation between two groups at 6-month follow up time. For all regression modelling to explore the difference between arms, group status will be included as explanatory variables. Data transformation would be needed for skewed outcome variables.

For peer review only - http://bmjopen.bmj.com/site/about/guidelines.xhtml

Health economic evaluation

Economic evaluation will be completed primarily from a health service perspective but in addition from a societal perspective. A cost effectiveness and cost utility analysis of the treatment options will be conducted. This will include incremental cost effectiveness (ICER) of Qbtest versus usual care; and present cost effectiveness acceptability curves (CEACs) for the diagnostic / treatment options. CEACs enable a probabilistic visual interpretation of the health economic analysis that can be used by decision makers to assist in their choice of health service delivery.

Implementation

To assess feasibility and acceptability we shall look at scores on the QbTest feedback questionnaires. High scores will be taken to indicate high acceptability and feasibility. Mean scores for individual items on each questionnaire will be assessed to determine which aspects of QbTest are perceived negatively or positively by clinicians and service users. Data from clinicians and patients who participate in interviews will be thematically analysed according to the principles of Braun and Clarke[44] to assess themes on the acceptability of QbTest, including patients' opinion on reduced length or number of clinic visits.

Data Monitoring

No interim analysis or analyses for safety or efficacy are planned.. Access to data will be restricted to trial team members and associated regulatory authorities as indicated in the sponsor agreement between sites and individual participant information sheets. The Chief Investigator (CH) shall oversee study management, with oversight from the rest of the research team. A sample (10% of the data) will be checked on a regular basis for verification of all entries made. Where corrections are required these will carry a full audit trail and justification, independent from the research team. There are no anticipated adverse effects of the QbTest, all adverse events will be recorded and monitored and the Chief Investigator (CH) will determine seriousness and causality and report the event to the ethics committee. The trial is overseen by an independent CLAHRC East Midlands Scientific Committee. The members of the committee are drawn externally from outside the institutions of the research team members and the trial sponsor.

For peer review only - http://bmjopen.bmj.com/site/about/guidelines.xhtml

ETHICS AND DISSEMINATION

The study received ethical approval (protocol version 1.2; 05.06.2014) from NRES Committee West Midlands – Coventry and Warwick (14/WM/0166) and has currently received local R&D approval from Alder Hey Children's NHS Foundation Trust, Medway NHS Foundation Trust, Central Manchester University Hospitals NHS Foundation Trust and Sussex Partnership NHS Trust. Patient recruitment has started at these sites and additional R&D approvals at other sites are in progress. The study is sponsored by the University Of Nottingham; neither the sponsor nor the funders will be involved in the analysis of study data or report writing. QbTech will provide QbTest reports to the study team, which will be analysed by BG, from the University Of Nottingham. Only the research team will have access to the study data, data generated from the trial will be available for inspection by the ethics and R&D committees upon request. Changes to the protocol will be communicated to the ethics committee by the lead research fellow (CLH). The process for obtaining participant informed consent or assent and parent / guardian informed consent will be in accordance with the ethical guidance, and Good Clinical Practice (GCP). The investigator or their nominee and the participant or other legally authorised representative (such as the child's parent) shall both sign and date the informed consent forms (Appendix A & B) before the person can participate in the study. Written consent will be required from young people aged 16-years and over and their parents. If the young person is under 16-years of age, parental consent will be required, with the young person's written or verbal assent. Individual participant medical information obtained as a result of this study are considered confidential and disclosure to third parties is prohibited unless warranted by an adverse event. Participant confidentiality will be further ensured by utilising identification code numbers to correspond to treatment data in the computer files. No post-trial care is required.

The primary aim of this study is to determine whether using QbTest in routine NHS settings can accelerate time to correct diagnosis, with a secondary aim of examining whether the QbTest can improve patient outcome. Currently, there are few trials conducted in routine NHS settings with the aim of improving the ADHD care pathway, despite evidence to suggest suboptimal care standards and rising socio-economic burdens. The findings of this study will help to demonstrate whether the QbTest is clinically useful and financially viable in standard care. The findings of the trial will be submitted for publication in appropriate journals regardless of outcome (in accordance with the recommendations of CONSORT) and to members of the public.

For peer review only

Acknowledgements: We would like to thank the site Principle Investigators: Adrian Williams (Alder Hey), Dr. Kim Selby (Medway), Dr. Samina Holsgrove (Central Manchester), Dr. Ify Omeneka (Warrington), Dr. Ann-Marie Skarstam (Sussex, Hastings), Dr. Sarah Curran (Sussex, Maidstone), Dr. Neeta Kulkarni (Leicester), Dr. Julie Clarke (Lincoln), Dr. Maria Moldavsky and Dr. Dilip Nathan (Nottingham) for their support. We would also like to thank: Teresa Jennings, Jo Dodd, Aaron Hobson, Sarah Murphy, Ann-Maria Regan, Susan Good, Gail Melvin and Julie Moss for facilitating the research at their respective sites. Finally, we would like to extend our thanks and appreciation to members of QbTech: Tony Doyle, Charlotte Keizer, Hans Bostrom and Fredrik Ulberstad for their advice and ongoing support.

Contributors: CH is the Chief Investigator and conceived and designed the study with the assistance of KS (co-investigator), DD (co-investigator), BG (medical statistician), and members of Qbtech (Hans Bostrom, Fredrik Ulberstad and Tony Doyle; co-investigators). CLH (lead research fellow) and CH wrote the protocol with approval from all authors. BG designed and wrote the statistical analysis plan. CLH, CH, AZV (researcher), GW (research assistant), CKH (clinical trials manager), KS and MJ (co-investigator) contributed to the drafting of the manuscript. All authors critically revised the manuscript for its important intellectual content. All authors read and approved the final manuscript.

Funding: The study is funded as part of the NIHR Collaborations in Leadership in Applied Health Research and Care (CLAHRC) East Midlands, funded by a central grant from the National Institute for Health Research (NIHR), with funding from QbTech in the form of QbTest systems and returning raw data back to the study sites (QbTest reports). The study sponsor and funders have no role in study design, including; collection, management, analysis and interpretation of data; writing of the report; and the decision to submit the report for publication.

Trial Sponsor: University of Nottingham, Mr Paul Cartledge: Kings Meadow Campus, Lenton Lane, Nottingham, NG7 2NR, sponsor@nottingham.ac.uk (REF: 14046).

Competing interests: None.

Ethics approval: This protocol was approved by NRES Committee West Midlands – Coventry and Warwick (14/WM/0166) on 11th July 2014.

Provenance and peer review: Not commissioned; externally peer reviewed.

REFERENCES

1. NICE. Attention deficit hyperactivity disorder: diagnosis and management of ADHD in children, young people and adults. *Clinical Guideline 72*. London: National Institute for Health and Clinical Excellence 2008.
2. Magyary D, Brandt, P. A decision tree and clinical paths for the assessment and management of children with ADHD. *Issues in mental health nursing* 2002; 23.6: 553-566.
3. Schlander M. Child and Adolescent Psychiatry and Mental Health. *Child and adolescent psychiatry and mental health* 2007;1(13):1-13.
4. King S, Griffin S, Hodges Z, Weatherly H, Asseburg C, Richardson G, et al. A systematic review and economic model of the effectiveness and cost-effectiveness of methylphenidate, dexamfetamine and atomoxetine for the treatment of attention deficit hyperactivity disorder in children and adolescents. *Health Technology Assessment (Winchester, England)* 2006;10(23):iii-iv, xiii-146.
5. D'Amico F, Knapp M, Beecham J, Sandberg S, Taylor E, Sayal K. Use of services and associated costs for young adults with childhood hyperactivity/conduct problems: 20-year follow-up. *The British Journal of Psychiatry* 2014; 204.6: 441-447.
6. Bolea-Alamañac B, Nutt DJ, Adamou M, Asherson P, Bazire S, Coghill D, et al.

   Evidence-based guidelines for the pharmacological management of attention deficit

For peer review only - http://bmjopen.bmj.com/site/about/guidelines.xhtml

hyperactivity disorder: Update on recommendations from the British Association for Psychopharmacology. *Journal of Psychopharmacology* 2014;28(3):179-203.

7. Ogundele MO, Ayyash HF, Banerjee S. Role of computerised continuous performance task tests in ADHD. *Progress in Neurology and Psychiatry* 2011;15(3):8-13.
8. Group MC. A 14-month randomized clinical trial of treatment strategies for attention-deficit/hyperactivity disorder. *Archives of General Psychiatry* 1999;56(12):1073.
9. McCarthy S, Cranswick N, Potts L, Taylor E, Wong IC. Mortality associated with attention-deficit hyperactivity disorder (ADHD) drug treatment. *Drug safety* 2009;32(11):1089-96.
10. Sonuga-Barke EJ, Sergeant JA, Nigg J, Willcutt E. Executive dysfunction and delay aversion in attention deficit hyperactivity disorder: nosologic and diagnostic implications. *Child and adolescent psychiatric clinics of North America* 2008;17(2):367-84.
11. Forbes GB. Clinical utility of the Test of Variables of Attention (TOVA) in the diagnosis of attention-deficit/hyperactivity disorder. *Journal of Clinical Psychology* 1998;54(4):461-76.
12. Sandford J, Turner A. Integrated visual and auditory continuous performance test manual. *Richmond, VA: BrainTrain* 2002.
13. Riccio CA, Cohen MJ, Hynd GW, Keith RW. Validity of the auditory continuous performance test in differentiating central processing auditory disorders with and without ADHD. *Journal of Learning Disabilities* 1996;29(5):561-66.
14. Conners CK, Staff MHS. Conners' Continuous Performance Test II (CPT II V. 5). *North Tonawanda, NY: Multi-Health Systems Inc* 2000.

For peer review only

For peer review only - http://bmjopen.bmj.com/site/about/guidelines.xhtml

15. Vogt C, Williams T. Early identification of stimulant treatment responders, partial responders and non-responders using objective measures in children and adolescents with hyperkinetic disorder. *Child and Adolescent Mental Health* 2011;16(3):144-49.

16. Losier BJ, McGrath PJ, Klein RM. Error patterns on the continuous performance test in non-medicated and medicated samples of children with and without ADHD: a meta-analytic review. *Journal of Child Psychology & Psychiatry & Allied Disciplines* 1996;37(8):971-87.

17. Riccio CA, Waldrop JJ, Reynolds CR, Lowe P. Effects of stimulants on the continuous performance test (CPT): implications for CPT use and interpretation. *Journal of Neuropsychiatry & Clinical Neurosciences* 2001;13(3):326-35.

18. Fernandez-Jaen A, Fernandez-Mayoralas DM, Pardos A, Calleja-Perez B, Munoz Jareno N. Clinical and cognitive response to extended-release methylphenidate (Medikinet) in attention deficit/hyperactivity disorder: efficacy evaluation. *Advances in Therapy* 2009;26(12):1097-110.

19. Huang Y-S, Chao C-C, Wu Y-Y, Chen Y-Y, Chen C-K. Acute effects of methylphenidate on performance during the Test of Variables of Attention in children with attention deficit/hyperactivity disorder. *Psychiatry and Clinical Neurosciences* 2007;61(3):219-25.

20. Solanto M, Newcorn J, Vail L, Gilbert S, Ivanov I, Lara R. Stimulant drug response in the predominantly inattentive and combined subtypes of attention-deficit/hyperactivity disorder. *Journal of Child and Adolescent Psychopharmacology* 2009;19(6):663-71.

21. Ulberstad F. QbTest Technical Manual. *Stockholm, Sweden: Qbtech AB* 2012.

For peer review only

22. Reh V, Schmidt M, Lam L, Schimmelmann BG, Hebebrand J, Rief W, et al. Behavioral Assessment of Core ADHD Symptoms Using the QbTest. *Journal of Attention Disorders* 2013: Doi 1087054712472981.

23. Reh v, Schidt M, Rief W, Christiansen H. Preliminary evidence for altered motion tracking-based hyperactivity in ADHD siblings. *Behavioral and Brain Functions 2014;* 10: DOI:10.1186/1744-9081-10-7.

24. Wehmeier PM, Schacht A, Wolff C, Otto WR, Dittmann RW, Banaschewski T. Neuropsychological outcomes across the day in children with attention-deficit/hyperactivity disorder treated with Atomoxetine: results from a placebo-controlled study using a computer-based continuous performance test combined with an infra-red motion-tracking device. *Journal of Child & Adolescent Psychopharmacology* 2011;21(5):433-44.

25. Ramtvedt BE, Ronias E, Aabech HS, Sundet KS. Clinical gains from including both dextroamphetamine and methylphenidate in stimulant trials. *Journal of child and adolescent psychopharmacology* 2013; 23: 597-604.

26. Günther T, Kahraman-Lanzerath B, Knospe EL, Herpertz-Dahlmann B, Konrad K.Modulation of Attention-Deficit/Hyperactivity Disorder Symptoms by Short-and Long-Acting Methylphenidate over the Course of a Day. *Journal of child and adolescent psychopharmacology* 2012; 22(2): 131-138.

27. Vogt C, Shameli A. Assessments for attention-deficit hyperactivity disorder: Use of objective measurements. *The Psychiatrist* 2011;35(10):380-83.

28. Oades RD, Myint A-M, Dauvermann MR, Schimmelmann BG, SCharz M. Attention-deficit hyperactivity disorder (ADHD) and glial integrity: an exploration of associations of cytokines and kynurenine metabolites with symptoms and attention. *Behavioural and Brain Functions 2010;*6 :32: 1-19.

For peer review only

29. Goodman R, Ford T, Richards H, Gatward R, Meltzer H. The Development and Well-Being Assessment: description and initial validation of an integrated assessment of child and adolescent psychopathology. *Journal of Child Psychology and Psychiatry* 2000;41(05):645-55.

30. Goodman R. The Strengths and Difficulties Questionnaire: a research note. *Journal of Child Psychology and Psychiatry* 1997;38(5):581-86.

31. Hill P, Taylor E. An auditable protocol for treating attention deficit/hyperactivity disorder. *Archives of disease in childhood* 2001;84(5):404-09.

32. Swanson JM, Sandman CA, Deutsch C, Baren M. Methylphenidate hydrochloride given with or before breakfast: I. Behavioral, cognitive, and electrophysiologic effects. *Pediatrics* 1983;72(1):49-55.

33. Shaffer D, Gould MS, Brasic J, Ambrosini P, Fisher P, Bird H, et al. A children's global assessment scale (CGAS). *Archives of General Psychiatry* 1983;40(11):1228-31.

34. Wille N, Badia X, Bonsel G, Burström K, Cavrini G, Devlin N, et al. Development of the EQ-5D-Y: a child-friendly version of the EQ-5D. *Quality of Life Research* 2010;19(6):875-86.

35. Beecham J, Knapp M. *Costing psychiatric interventions*: Gaskell In Measuring Mental Health Needs. Edited by Thornicroft G. London, 2001.

36. Feiveson A. Power by simulation. *Stata J* 2002;2(2):107-24.

37. Van Belle G. *Statistical rules of thumb*: John Wiley & Sons, 2011.

38. Smeeth L, Ng ES-W. Intraclass correlation coefficients for cluster randomized trials in primary care: data from the MRC Trial of the Assessment and Management of Older People in the Community. *Controlled clinical trials* 2002;23(4):409-21.

For peer review only

39. Elley CR, Kerse N, Chondros P, Robinson E. Intraclass correlation coefficients from three cluster randomised controlled trials in primary and residential health care. *Australian and New Zealand journal of public health* 2005;29(5):461-67.
40. Sayal K, Daley D, James M, Yang M, Batty MJ, Taylor JA, et al. Protocol Evaluating the effectiveness of a school-based group programme for parents of children at risk of ADHD: the 'PArents, Teachers and CHildren WORKing Together (PATCHWORK)'cluster RCT protocol. *BMJ open* 2012;2(5). Doi:10.1136/bmjopen-2012-001783
41. Goldstein H. *Multilevel statistical models*: John Wiley & Sons, 2011.
42. Dinnes J, Deeks J, Kirby J, Roderick P. A methodological review of how heterogeneity has been examined in systematic reviews of diagnostic test accuracy. *Health Technology Assessment* 2005;9(12).
43. Mallett S, Halligan S, Thompson M, Collins GS, Altman DG. Interpreting diagnostic accuracy studies for patient care. *BMJ: British Medical Journal* 2012;345.
44. Braun V, Clarke V. Using thematic analysis in psychology. *Qualitative research in psychology* 2006;3(2):77-101.

For peer review only

Figure 1: Study Flowchart

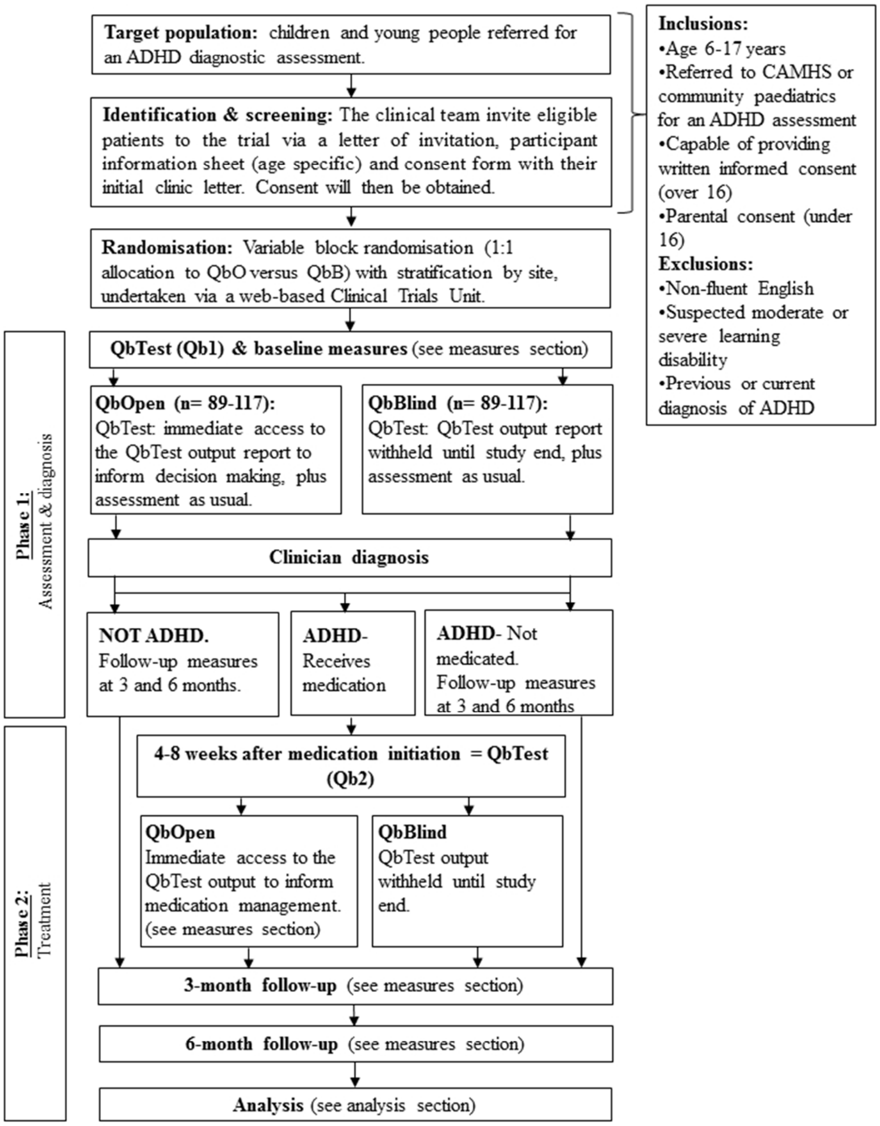

190x254mm (96 x 96 DPI)

The University of Nottingham
UNITED KINGDOM · CHINA · MALAYSIA

Alder Hey Children's NHS
NHS Foundation Trust

CONSENT FORM FOR PARENTS
(Final version 1.2: 05.06.2014)

Title of Study: Assessing QbTest Utility in ADHD: A Randomised Controlled Trial

REC ref: *14/WM/0166*

Name of Researcher:

Name of Participant (Parent):

Name of Participant (Child):

Please INITIAL box

1. I confirm that I have read and understand the information sheet version number 1.2 dated 05.06.2014 for the above study and have had the opportunity to ask questions. ☐

2. I understand that mine and my child's participation is voluntary and that we are free to withdraw at any time, without giving any reason, and without their medical care or legal rights being affected. I understand that should we withdraw then the information collected so far cannot be erased and that this information may still be used in the project analysis. ☐

3. I understand that relevant sections of my child's medical notes and data collected in the study may be looked at by authorised individuals from the University of Nottingham, the research group and regulatory authorities where it is relevant to our taking part in this study. I give permission for these individuals to have access to these records and to collect, store, analyse and publish information obtained from mine and my child's participation in this study. I understand that mine and my child's personal details will be kept confidential. ☐

4. I agree for my child's teacher and head teacher to be contacted and be asked to complete questionnaires about my child's behaviour at school. ☐

5. I understand if I/my child take part in any research interviews, they will be recorded and that anonymous direct quotes from the focus group may be used in the study reports. ☐

6. I agree for me and my child (named above) to take part in the above study. ☐

Name of Parent	Date	Signature

<u>(OPTIONAL) Section for children to give assent</u>

I agree to take part in this study

Name of Child (for assent)	Date	Signature

For peer review only - http://bmjopen.bmj.com/site/about/guidelines.xhtml

The University of Nottingham
UNITED KINGDOM · CHINA · MALAYSIA

Alder Hey Children's NHS
NHS Foundation Trust

To be completed by researcher/investigator taking consent:

Researcher consent	Date	Signature

For peer review only

For peer review only - http://bmjopen.bmj.com/site/about/guidelines.xhtml

Appendix B

CONSENT FORM FOR YOUNG PERSON

(Final version 1.2: 05.06.2014)

Title of Study: Assessing QbTest Utility in ADHD: A Randomised Controlled Trial

REC ref: ***14/WM/0166***

Name of Researcher:

Name of Participant (young person):

Name of Participant (parent):

Please INITIAL box

1. I confirm that I have read and understand the information sheet version number 1.2 dated 05.06.2014 for the above study and have had the opportunity to ask questions. ☐
2. I understand that my participation is voluntary and that I am free to withdraw at any time, without giving any reason, and without their medical care or legal rights being affected. I understand that should they withdraw then the information collected so far cannot be erased and that this information may still be used in the project analysis. ☐
3. I understand that relevant sections of my medical notes and data collected in the study may be looked at by authorised individuals from the University of Nottingham, the research group and regulatory authorities where it is relevant to our taking part in this study. I give permission for these individuals to have access to these records and to collect, store, analyse and publish information obtained from my child's participation in this study. I understand that my child's personal details will be kept confidential. ☐
4. I agree for my teacher and head teacher to be contacted and be asked to complete questionnaires about my behaviour at school. ☐
5. I understand if I/ take part in any research interviews, they will be recorded and that anonymous direct quotes from the focus group may be used in the study reports. ☐
6. I agree to take part in the above study. ☐

Name of young person	Date	Signature

To be completed by researcher/investigator taking consent:

Name of researcher	Date	Signature

For peer review only - http://bmjopen.bmj.com/site/about/guidelines.xhtml

Assessing QbTest Utility in ADHD: A Randomised Controlled Trial V1.2 05.06.2014

For peer review only

SPIRIT
STANDARD PROTOCOL ITEMS: RECOMMENDATIONS FOR INTERVENTIONAL TRIALS

SPIRIT 2013 Checklist: Recommended items to address in a clinical trial protocol and related documents*

Section/item	Item No	Description
**Administrative information**		
√ Title	1	Descriptive title identifying the study design, population, interventions, and, if applicable, trial acronym
√ Trial registration	2a	Trial identifier and registry name. If not yet registered, name of intended registry
	2b	All items from the World Health Organization Trial Registration Data Set
√ Protocol version	3	Date and version identifier
√ Funding	4	Sources and types of financial, material, and other support
√ Roles and responsibilities	5a	Names, affiliations, and roles of protocol contributors
	5b	Name and contact information for the trial sponsor
	5c	Role of study sponsor and funders, if any, in study design; collection, management, analysis, and interpretation of data; writing of the report; and the decision to submit the report for publication, including whether they will have ultimate authority over any of these activities
	5d	Composition, roles, and responsibilities of the coordinating centre, steering committee, endpoint adjudication committee, data management team, and other individuals or groups overseeing the trial, if applicable (see Item 21a for data monitoring committee)
**Introduction**		
√ Background and rationale	6a	Description of research question and justification for undertaking the trial, including summary of relevant studies (published and unpublished) examining benefits and harms for each intervention
	6b	Explanation for choice of comparators
√ Objectives	7	Specific objectives or hypotheses

For peer review only

√ Trial design	8	Description of trial design including type of trial (eg, parallel group, crossover, factorial, single group), allocation ratio, and framework (eg, superiority, equivalence, noninferiority, exploratory)

Comment [UoN1]: We have chosen to put this in the methods section, if you prefer we will move it to the introduction, but we feel it flows better as the start of the method

Methods: Participants, interventions, and outcomes

√ Study setting	9	Description of study settings (eg, community clinic, academic hospital) and list of countries where data will be collected. Reference to where list of study sites can be obtained
√ Eligibility criteria	10	Inclusion and exclusion criteria for participants. If applicable, eligibility criteria for study centres and individuals who will perform the interventions (eg, surgeons, psychotherapists)
√ Interventions	11a	Interventions for each group with sufficient detail to allow replication, including how and when they will be administered
	11b	Criteria for discontinuing or modifying allocated interventions for a given trial participant (eg, drug dose change in response to harms, participant request, or improving/worsening disease)
	11c	Strategies to improve adherence to intervention protocols, and any procedures for monitoring adherence (eg, drug tablet return, laboratory tests)
	11d	Relevant concomitant care and interventions that are permitted or prohibited during the trial
√ Outcomes	12	Primary, secondary, and other outcomes, including the specific measurement variable (eg, systolic blood pressure), analysis metric (eg, change from baseline, final value, time to event), method of aggregation (eg, median, proportion), and time point for each outcome. Explanation of the clinical relevance of chosen efficacy and harm outcomes is strongly recommended
√ Participant timeline	13	Time schedule of enrolment, interventions (including any run-ins and washouts), assessments, and visits for participants. A schematic diagram is highly recommended (see Figure)
√ Sample size	14	Estimated number of participants needed to achieve study objectives and how it was determined, including clinical and statistical assumptions supporting any sample size calculations
√ Recruitment	15	Strategies for achieving adequate participant enrolment to reach target sample size

Methods: Assignment of interventions (for controlled trials)

Allocation:

For peer review only - http://bmjopen.bmj.com/site/about/guidelines.xhtml

√ Sequence generation	16a	Method of generating the allocation sequence (eg, computer-generated random numbers), and list of any factors for stratification. To reduce predictability of a random sequence, details of any planned restriction (eg, blocking) should be provided in a separate document that is unavailable to those who enrol participants or assign interventions
√ Allocation concealment mechanism	16b	Mechanism of implementing the allocation sequence (eg, central telephone; sequentially numbered, opaque, sealed envelopes), describing any steps to conceal the sequence until interventions are assigned
√Implementati on	16c	Who will generate the allocation sequence, who will enrol participants, and who will assign participants to interventions
√ Blinding (masking)	17a	Who will be blinded after assignment to interventions (eg, trial participants, care providers, outcome assessors, data analysts), and how
	17b	If blinded, circumstances under which unblinding is permissible, and procedure for revealing a participant's allocated intervention during the trial
**Methods: Data collection, management, and analysis**		
√ Data collection methods	18a	Plans for assessment and collection of outcome, baseline, and other trial data, including any related processes to promote data quality (eg, duplicate measurements, training of assessors) and a description of study instruments (eg, questionnaires, laboratory tests) along with their reliability and validity, if known. Reference to where data collection forms can be found, if not in the protocol
	18b	Plans to promote participant retention and complete follow-up, including list of any outcome data to be collected for participants who discontinue or deviate from intervention protocols
√ Data management	19	Plans for data entry, coding, security, and storage, including any related processes to promote data quality (eg, double data entry; range checks for data values). Reference to where details of data management procedures can be found, if not in the protocol
√ Statistical methods	20a	Statistical methods for analysing primary and secondary outcomes. Reference to where other details of the statistical analysis plan can be found, if not in the protocol
	20b	Methods for any additional analyses (eg, subgroup and adjusted analyses)
	20c	Definition of analysis population relating to protocol non-adherence (eg, as randomised analysis), and any statistical methods to handle missing data (eg, multiple imputation)

Methods: Monitoring

√ Data monitoring	21a	Composition of data monitoring committee (DMC); summary of its role and reporting structure; statement of whether it is independent from the sponsor and competing interests; and reference to where further details about its charter can be found, if not in the protocol. Alternatively, an explanation of why a DMC is not needed
	21b	Description of any interim analyses and stopping guidelines, including who will have access to these interim results and make the final decision to terminate the trial
√ Harms	22	Plans for collecting, assessing, reporting, and managing solicited and spontaneously reported adverse events and other unintended effects of trial interventions or trial conduct
√ Auditing	23	Frequency and procedures for auditing trial conduct, if any, and whether the process will be independent from investigators and the sponsor

Ethics and dissemination

√ Research ethics approval	24	Plans for seeking research ethics committee/institutional review board (REC/IRB) approval
√ Protocol amendments	25	Plans for communicating important protocol modifications (eg, changes to eligibility criteria, outcomes, analyses) to relevant parties (eg, investigators, REC/IRBs, trial participants, trial registries, journals, regulators)
√ Consent or assent	26a	Who will obtain informed consent or assent from potential trial participants or authorised surrogates, and how (see Item 32)
	26b	Additional consent provisions for collection and use of participant data and biological specimens in ancillary studies, if applicable
√ Confidentiality	27	How personal information about potential and enrolled participants will be collected, shared, and maintained in order to protect confidentiality before, during, and after the trial
√ Declaration of interests	28	Financial and other competing interests for principal investigators for the overall trial and each study site
√ Access to data	29	Statement of who will have access to the final trial dataset, and disclosure of contractual agreements that limit such access for investigators
√ Ancillary and post-trial care	30	Provisions, if any, for ancillary and post-trial care, and for compensation to those who suffer harm from trial participation

For peer review only - http://bmjopen.bmj.com/site/about/guidelines.xhtml

√Dissemination policy	31a	Plans for investigators and sponsor to communicate trial results to participants, healthcare professionals, the public, and other relevant groups (eg, via publication, reporting in results databases, or other data sharing arrangements), including any publication restrictions
	31b	Authorship eligibility guidelines and any intended use of professional writers
	31c	Plans, if any, for granting public access to the full protocol, participant-level dataset, and statistical code
**Appendices**		
√ Informed consent materials	32	Model consent form and other related documentation given to participants and authorised surrogates
X Biological specimens	33	Plans for collection, laboratory evaluation, and storage of biological specimens for genetic or molecular analysis in the current trial and for future use in ancillary studies, if applicable

Comment [UoN2]: Not relevant

*It is strongly recommended that this checklist be read in conjunction with the SPIRIT 2013 Explanation & Elaboration for important clarification on the items. Amendments to the protocol should be tracked and dated. The SPIRIT checklist is copyrighted by the SPIRIT Group under the Creative Commons "Attribution-NonCommercial-NoDerivs 3.0 Unported" license.

For peer review only - http://bmjopen.bmj.com/site/about/guidelines.xhtml

Figure 1: Study Flowchart

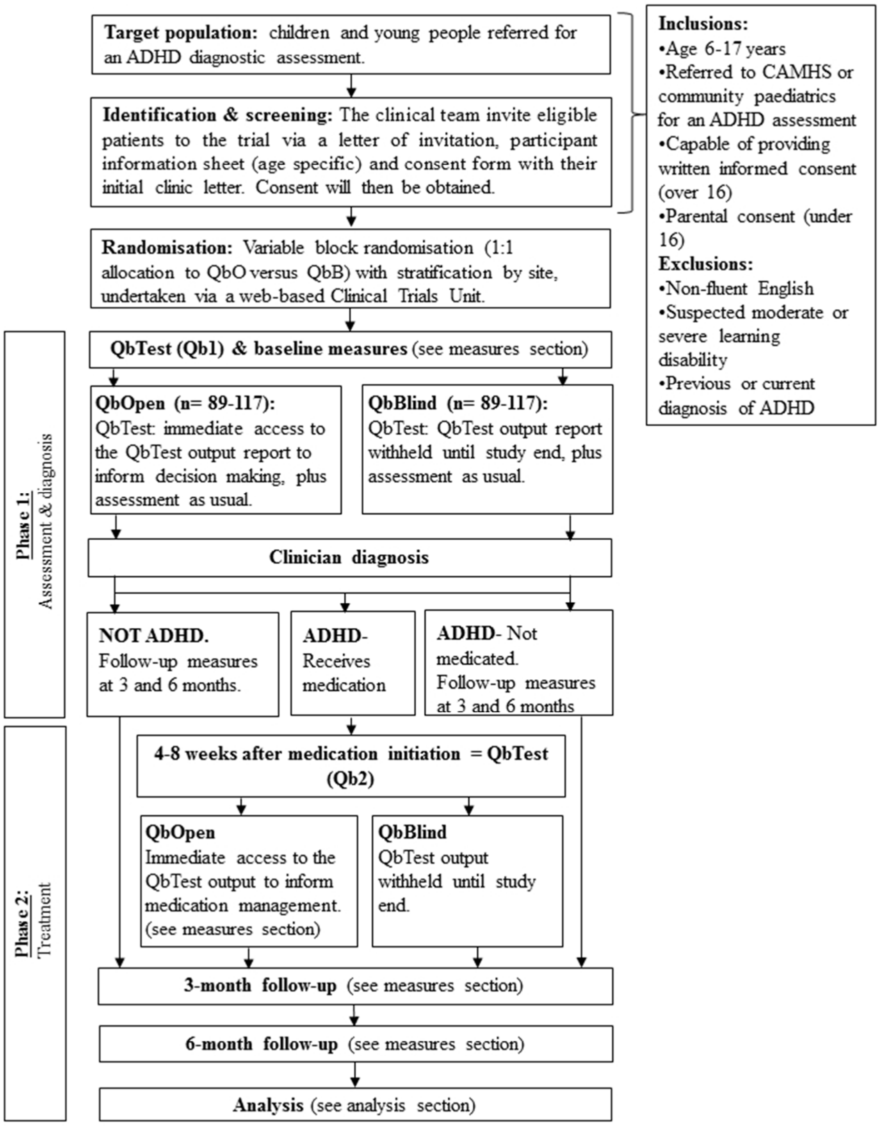

190x254mm (96 x 96 DPI)

The University of Nottingham
UNITED KINGDOM · CHINA · MALAYSIA

Appendix B

CONSENT FORM FOR YOUNG PERSON

(Final version 1.2: 05.06.2014)

Title of Study: Assessing QbTest Utility in ADHD: A Randomised Controlled Trial

REC ref: ***14/WM/0166***

Name of Researcher:

Name of Participant (young person):

Name of Participant (parent):

Please INITIAL box

1. I confirm that I have read and understand the information sheet version number 1.2 dated 05.06.2014 for the above study and have had the opportunity to ask questions. ☐
2. I understand that my participation is voluntary and that I am free to withdraw at any time, without giving any reason, and without their medical care or legal rights being affected. I understand that should they withdraw then the information collected so far cannot be erased and that this information may still be used in the project analysis. ☐
3. I understand that relevant sections of my medical notes and data collected in the study may be looked at by authorised individuals from the University of Nottingham, the research group and regulatory authorities where it is relevant to our taking part in this study. I give permission for these individuals to have access to these records and to collect, store, analyse and publish information obtained from my child's participation in this study. I understand that my child's personal details will be kept confidential. ☐
4. I agree for my teacher and head teacher to be contacted and be asked to complete questionnaires about my behaviour at school. ☐
5. I understand if I/ take part in any research interviews, they will be recorded and that anonymous direct quotes from the focus group may be used in the study reports. ☐
6. I agree to take part in the above study. ☐

Name of young person	Date	Signature

To be completed by researcher/investigator taking consent:

Name of researcher	Date	Signature

For peer review only

The University of Nottingham
UNITED KINGDOM · CHINA · MALAYSIA

For peer review only

Assessing QbTest Utility in ADHD: A Randomised Controlled Trial V1.2 05.06.2014

BMJ Open

Protocol investigating the clinical utility of an objective measure of activity and attention (QbTest) on diagnostic and treatment decision-making in children and young people with ADHD: 'Assessing QbTest Utility in ADHD' (AQUA) a randomised controlled trial

Journal:	*BMJ Open*
Manuscript ID:	bmjopen-2014-006838.R1
Article Type:	Protocol
Date Submitted by the Author:	06-Nov-2014
Complete List of Authors:	Hall, Charlotte; Institute of Mental Health, NIHR CLAHRC-East Midlands, Psychiatry<br>Walker, Gemma; Institute of Mental Health, Psychiatry<br>valentine, althea; University of Nottingham,<br>Guo, Boliang; Institute of Mental Health, Psychiatry<br>Kaylor-Hughes, Catherine; Institute of Mental Health, Psychiatry<br>james, marilyn; Institute of Mental Health, Psychiatry; University of Nottingham,<br>daley, david; Institute of Mental Health, Psychiatry<br>Sayal, Kapil; Institute of Mental Health, Psychiatry; University of Nottingham,<br>Hollis, Chris; Institute of Mental Health, Psychiatry; University of Nottingham, Division of Psychiatry
**Primary Subject Heading**:	Mental health
Secondary Subject Heading:	Health economics, Paediatrics
Keywords:	ADHD, Continuous Performance Test (CPT), QbTest, Diagnosis, Treatment

SCHOLARONE™
Manuscripts

For peer review only

Protocol investigating the clinical utility of an objective measure of activity and attention (QbTest) on diagnostic and treatment decision-making in children and young people with ADHD: 'Assessing QbTest Utility in ADHD' (AQUA) a randomised controlled trial

Authors

[1]Charlotte L. Hall, Research Fellow, CLAHRC, University of Nottingham, Nottingham, UK

[2]Gemma M. Walker, Research Assistant, CLAHRC, University of Nottingham, Nottingham, UK

[3]Althea Z. Valentine, Researcher, CLAHRC, University of Nottingham, Nottingham, UK

[4]Boliang Guo, Medical Statistician, CLAHRC, University of Nottingham, Nottingham, UK

[5]Catherine Kaylor-Hughes, Clinical Trials Manager, CLAHRC, University of Nottingham, Nottingham, UK

[6]Marilyn James, Professor of Health Economics, Division of Rehabilitation and Ageing, University of Nottingham, Nottingham, UK

[7]David Daley, Professor of Psychological Intervention and Behaviour Change, Division of Psychiatry & Applied Psychology, School of Medicine, University of Nottingham, Nottingham, UK

[8]Kapil Sayal, Professor of Child & Adolescent Psychiatry, Developmental Psychiatry, University of Nottingham, Queens Medical Centre, Nottingham, UK

[9]Chris Hollis, Professor of Child & Adolescent Psychiatry, Developmental Psychiatry, University of Nottingham, Queens Medical Centre, Nottingham, UK

Corresponding Author

[9]Chris Hollis,

Institute of Mental Health

University of Nottingham

Triumph Road

Nottingham, NG7 2TU

Chris.hollis@nottingham.ac.uk: 0115 8230258

Key words: ADHD, Continuous Performance Test (CPT), QbTest, diagnosis, treatment

Word count: 4738 (excluding title page, abstract, summary, references and figures)

For peer review only

ABSTRACT

Introduction

The National Institute for Health and Care Excellence (NICE) guidelines for attention deficit/hyperactivity disorder (ADHD) state that young people need to have access to the best evidence-based care to improve outcome. The current 'gold standard' ADHD diagnostic assessment combines clinical observation with subjective parent, teacher and self-reports. In routine practice, reports from multiple informants may be unavailable or contradictory, leading to diagnostic uncertainty and delay. The addition of objective tests of attention and activity may help reduce diagnostic uncertainty and delays in initiating treatment leading to improved outcomes. This trial investigates whether providing clinicians with an objective report of levels of attention, impulsivity and activity can lead to an earlier, and more accurate, clinical diagnosis and improved patient outcome.

Methods and analysis

This multi-site randomised controlled trial (RCT) will recruit young people (aged 6-17-years old) who have been referred for an ADHD diagnostic assessment at Child and Adolescent Mental Health Services (CAMHS) and Community Paediatric clinics across England. Routine clinical assessment will be augmented by the QbTest, incorporating a computerized continuous performance test (CPT) and infrared motion tracking of activity. The participant will be randomised into one of two study arms: QbOpen (clinician has immediate access to a QbTest report): QbBlind (report is withheld until the study end). Primary outcomes are time to diagnosis and diagnostic accuracy. Secondary outcomes include clinician's diagnostic confidence and routine clinical outcome measures. Cost-effective analysis will be conducted, alongside a qualitative assessment of the feasibility and acceptability of incorporating QbTest in routine practice.

Ethics and dissemination

The findings from the study will inform commissioners, clinicians and managers about the feasibility, acceptability, clinical utility and cost effectiveness of incorporating QbTest into routine diagnostic assessment of young people with ADHD. The results will be submitted for publication in peer-reviewed journals. The study has received ethical approval.

Trial registration: NCT02209116

For peer review only

INTRODUCTION

Attention Deficit/Hyperactivity Disorder (ADHD) is the most common neuro-developmental disorder and affects 3-5% of children and young people[1]. The core symptoms of ADHD include poor attention, hyper-activity and impulsivity. National Institute for Health and Care Excellence (NICE)[1] guidelines provide a blueprint for the diagnosis and management of ADHD in England and Wales and indicate the need for young people with ADHD to have access to the best evidence-based care in order to fulfil their potential and prevent poor outcome. However, in practice, delivery and quality of care is ad-hoc, with little consistency in assessment, diagnosis or management[2]. ADHD frequently co-exists with other neurodevelopmental and psychiatric disorders and is a risk factor for major educational, social and occupational impairment, placing a huge burden on the NHS, social care and criminal justice systems. There has been a rapid growth in diagnosis over the last 30 years with the number of children recognised and treated for ADHD in the UK increasing almost 10 fold from the early 1980s[1], and spending on medication for ADHD increasing seven fold between 1998 and 2005[3]. The cost of initial specialist assessment for ADHD is estimated at £23 million annually in England and Wales[4], and drug costs for ADHD in England during 2012 was expected to exceed £78 million[3], while indirect costs to families include parental mental ill health, time off work and loss of earnings are even higher[5]. Increasing recognition of ADHD as a lifespan condition is placing a new demand on the NHS to provide diagnostic and treatment services for children, adolescents and adults, exposing serious limitations in existing methods of assessment and management.

There is no single test, or biomarker used to diagnose the disorder[6]. In the absence of any objective measure to identify ADHD, clinical assessment and diagnosis is based on the clinician's integration of various forms of subjective information including direct observation and reports from parents, teachers and young people. This approach is heavily reliant on subjective measures and clinical interpretation, which can lead to lack of reliability and consistency in the diagnosis of ADHD[7] and furthermore, the process of 'gold standard' clinical interviews and data collection from multiple informants is time consuming and often

For peer review only - http://bmjopen.bmj.com/site/about/guidelines.xhtml

difficult to conduct in real world settings with frequent missing data and inconsistencies between reports leading to and diagnostic uncertainty and delay.

Additionally, whilst treatments for ADHD are highly efficacious in carefully managed research settings[1], in standard community care the outcome of treatment may be sub-optimal. Aside from delays in initiating treatment caused by diagnostic uncertainty, once on medication, children may not be reviewed sufficiently frequently for clinicians to detect non- or partial-response, or to establish the optimal dose for each child. The U.S. National Institute of Mental Health (NIMH) Multimodal Treatment study of ADHD (MTA) showed that careful medication management can significantly improve outcomes, doubling the normalisation rate from 25% in routine community care to almost 60% when using a strategy of careful dose titration and frequent monitoring of outcome[8]. The NICE[1] ADHD guidelines recommends that during the titration phase, symptoms should be closely monitored using rating scales. However, audit data within the East Midlands showed that community care for ADHD falls well below the standards for titration and monitoring set out in the MTA and NICE guidelines (CLAHRC-NDL, 2013, unpublished audit). A further consequence of sub-optimal treatment response in routine care is poor medication adherence. In the U.K., 50% of patients have stopped ADHD medication after 18 months and 80% after 3 years[9].

Objective Assessment Measures in ADHD

One approach to improving assessment and outcomes in routine care is to add objective laboratory measures of activity and attention for both diagnostic assessment and treatment optimisation[5]. Objective measures have the potential to augment and streamline current practice in order to shorten assessment time, increase diagnostic accuracy, reduce delays in treatment, and optimise treatment response.

Continuous Performance Test

A Continuous Performance Test (CPT) is a neuropsychological test that measures the individual's capacity to sustain attention (vigilance) and inhibit inappropriate responses (impulsivity), which can be used alongside clinical evaluation to inform the diagnostic process[10]. Typically, a CPT is a computer based programme which involves rapid presentation of visual or auditory stimuli. Participants are asked to respond when a given target occurs but remain passive to non-targets. A standard CPT typically records the child's

For peer review only

omission errors (responding when the target is present), commission errors (responding when the target is not present), number of correct responses, reaction time and variability in reaction time. There are several well validated commercially available CPT tests such as the TOVA (Test of Variables of Attention[11]), IVA (Integrated Visual and Auditory[12]), ACPT (Auditory Continuous Performance Test[13]) and the Conner's CPT[14]. These tests are one of the most popular clinic based measures to assess sustained attention in children[15]; with several studies and a meta-analyses showing that children with ADHD perform worse on these tasks than children without ADHD[16]. CPTs have also been shown to be a sensitive measure of medication effects[17]. Several studies have noted improvement in CPT scores in children with ADHD on stimulant medication[15, 18-20]. However, little research has compared CPT scores with more subjective measurements of ADHD[7] and in their recent review Ogundele et al.[7] recommended further research in the use of CPTs compared to standard practice to determine cost effectiveness of these tasks.

A significant limitation of the CPT for the assessment of ADHD is that it doesn't measure the patients' activity levels, which is a core symptom domain of ADHD. Approaches to the objective measurement of activity in ADHD have included wrist-worn actigraphy devices and infra-red motion capture.

QbTest

QbTest (Qbtech Ltd) has been developed to combine a CPT to measure attention and impulsivity with infra-red motion capture of head movement during the CPT to measure activity [21]. The QbTest CPT requires participants to respond to an infrequently presented stimulus (by pressing a button) but ignore all others. Physical activity is measured during the course of the CPT via an infra-red camera that tracks the path of a reflector attached to the participants head (central midpoint). These elements of the test provide information on each of the three symptom domains of ADHD and provide summary scores for each individual based on deviation from a normative dataset based on age group and gender. There are two versions of the task for children and young people; the task for 6-11-year olds is 15 minutes duration and the task for 12-17-year olds is 20 minutes duration. The QbTest result is complemented by a clinical evaluation and behavioural observation of events that may affect test performance. The QbTest is not a stand-alone diagnostic tool, but has been approved by the U.S. Food and Drug Administration (FDA; Ref: K133382) to supplement standard

For peer review only - http://bmjopen.bmj.com/site/about/guidelines.xhtml

clinical assessment and treatment follow-up by reducing reliance on measures such as subjective observer reports (which can be biased, incomplete or missing) and augment clinical decision-making.

The data available about the QbTest has shown favourable psychometric characteristics with child participants. For example, Qbtech report data that establishes 85% sensitivity of classification and 92% specificity, and good test-retest scores, which is not influenced by experience with computers (Ulberstad, 2012, unpublished data). Reh et al.[22] investigated the factor structure and concurrent and discriminant validity of QbTest and found the hyperactivity factor correlated with teacher ratings of hyperactive behaviour, providing evidence for the utility of including this additional measure of activity in a CPT. In addition, Reh et al.[23] found the hyperactivity factor could identify intermediate levels of impairment in ADHD siblings, suggesting this factor maybe particularly sensitivity as an intermediate phenotype for ADHD. Their findings also provide initial evidence for the concurrent validity of the three factors (attention, impulsivity, activity), although the authors highlight the need for further research to investigate validity. Wehmeier et al.[24] found QbTest to be a valid measure of treatment outcome and highly correlated with blinded observer ratings of behaviour in placebo-controlled RCTs. QbTest is effective in evaluating ADHD medication effects in children[25 26], and can identify early non-responders[15]. One clinical study found QbTest improved clinical accuracy by reducing the risk of unidentified ADHD when patients were re-evaluated one-year after their initial assessment[27] and another indicated the ability for QbTest to differentiate ADHD from normative controls[28]. Initial audit data (Selby, 2013, unpublished data) suggest that implementation of QbTest in routine ADHD clinics can reduce the time to diagnosis by 30%. This equates to a reduction from an average of three to two out-patient appointments per patient in order to either confirm or exclude a diagnosis of ADHD. These findings indicate potential for QbTest to support the diagnostic assessment and management of ADHD within routine clinical practice; however, there has been no randomised controlled trial (RCT) to investigate the added clinical value (clinical utility) and economic cost effectiveness of adding QbTest to standard ADHD care pathways within the NHS.

The primary aim of the Assessing QbTest Utility in ADHD-trial (AQUA-Trial) is to determine whether using QbTest in routine NHS settings can accelerate diagnosis without compromising diagnostic accuracy. Secondly, the study aims to examine whether QbTest

For peer review only - http://bmjopen.bmj.com/site/about/guidelines.xhtml

improves the medication titration process by increasing the proportion of patients normalised after 6 months post baseline assessment and improves patient outcome. The study will also use qualitative methods to explore the barriers, drivers and facilitators to the adoption of the QbTest in routine practice. The cost-effectiveness of implementing the QbTest in practice will also be investigated. The findings will indicate whether establishing QbTest as part of standard practice in ADHD assessment and management is clinically useful, financially viable and acceptable for clinicians and patients.

METHODS AND ANALYSIS

Trial Design

This study is a parallel group single-blind multi-center RCT, exploring feasibility and acceptability, using quantitative and qualitative and health economic evaluations. The study consists of two arms:

1) QbOpen (QbO)

In this arm of the trial participants will complete the QbTest and clinicians, participants and their families will have immediate access to a QbTest report.

2) QbBlind: (QbB):

In this arm of the trial participants will complete the QbTest but the QbTest report will be withheld from the clinician, participant and patient's family until the last outcome measure is completed at 6 months. All participants will receive the same intervention. Specifically, this will be assessment as usual plus a QbTest as part of the diagnostic assessment.

The patients usual care team will be responsible for conducting the QbTest in clinic appointments. The QbTest will be only be conducted by trained QbTest clinicians.

Setting

Child and Adolescent Mental Health Services (CAMHS) and Community Paediatric clinics across 9 different NHS Trusts in England, including Medway NHS Foundation Trust, Alder Hey Children's NHS Foundation Trust, Nottinghamshire Healthcare NHS Trust, Leicestershire Partnership NHS Trust, Sussex Partnership NHS Foundation Trust, United

For peer review only

Lincolnshire Hospital NHS Trust, Central Manchester University Hospitals NHS Foundation Trust, Bridgewater Community Healthcare NHS Trust, Nottingham University Hospitals NHS Trust. Additional NHS Trusts may be recruited to meet target recruitment figures.

Recruitment and eligibility

New referrals for a diagnostic evaluation for suspected ADHD will be invited to participate in the research based on the following criteria:

Inclusion criteria

- Age 6-17-years old (at the time of consent)
- Referred to CAMHS or Community Paediatrics for an ADHD diagnostic assessment
- Capable of providing written informed consent (over 16-years old)
- Parental consent (under 16-years old)

Exclusion criteria

- Severe learning disability (to be assessed by clinical judgment)
- Non-fluent English speaking
- Previous or current confirmed diagnosis of ADHD
- Currently receiving ADHD medication

Written information about the trial will be sent to families through the clinic administrators prior to their first appointment. Clinic invitations will be updated and recorded on a password protected database on a weekly basis. Parents and young people who wish to participate will be asked to complete and return a consent form before this appointment. Alternatively, participants will be consented into the study by the clinical team, Clinical Studies Officer or a member of the research team at their first appointment. Each site will be informed of the monthly recruitment target required in order to meet the study sample size and updated on their monthly progress.

Trial Phases

There are two phases to this study (see Figure 1).

For peer review only

Phase 1, Assessment: The first phase investigates the use of QbTest as a tool to aid diagnosis. Participants will undergo ADHD 'assessment as usual', which varies between clinics, clinicians and cases, but will typically involve an interview with the parent/young person and collection of questionnaires from the parent/young person and teacher. Whilst attending the clinic, the participant will be asked to complete the QbTest at some point during the diagnostic process. Participants will also be asked to complete baseline outcome measures (see measures section).

Phase 2, Treatment: Patients who receive a clinic diagnosis of ADHD and are allocated by clinicians to receive ADHD medication initiated within 3 months of their baseline assessment will be asked to complete a second QbTest (Qb2) 4-8 weeks after medication initiation. This timeframe was chosen to ensure that all participants can complete their second QbTest before the 6-month follow-up.

All participants will stay in the trial for 6-months and will be asked to complete outcome measures at 3 and 6-month follow up, regardless of their diagnosis or whether they receive medication. With the aim of promoting participant retention and completion of follow-up measures, participants will be compensated for their time with a £15 high-street voucher if they remain in the trial until 6-months.

Measures

Blinded members of the research team (CLH, GMW, AVZ,) will be fully trained in all trial assessments and responsible for monitoring the distribution, completion and collection of all outcome measures.

Primary Outcome

The primary outcome is the number of consultations until a confirmed clinical diagnosis is reached, as recorded on a short pro-forma. The pro-forma will be completed by clinicians after each consultation with the young person and/or family and documents information about appointment duration, diagnosis and medication/treatment. The pro-forma can be provided by contacting the corresponding author.

Secondary Outcomes

The secondary outcomes obtained from the pro-forma are:

For peer review only - http://bmjopen.bmj.com/site/about/guidelines.xhtml

- Number of days and duration of visits (in minutes) until a confirmed diagnosis is reached.
- Clinical confidence in diagnostic decision. Clinicians will be required to rate the confidence of their decision on a 7-point Likert scale (Definitely ADHD-Definitely not ADHD).
- Stability in diagnosis. Clinicians will be required to re-rate their diagnostic decision and confidence at 6-months.

Other measures

- Development and Well-being Assessment (DAWBA[29]): The DAWBA is a semi-structured, investigator-based diagnostic interview for child mental health problems, including ADHD, which includes the (Strengths and Difficulties Questionnaire; SDQ[30]) as an initial screen. The parent and teacher DAWBA will be completed to compare the accuracy of clinic diagnosis (in both QbO and QbB arms of the trial) to that of an independent clinical consensus diagnosis made using the DAWBA. Two experienced clinicians, blind to allocation, will review the DAWBA and arrive at a clinical consensus diagnosis.
- QbTest scores: Q-scores for attention, impulsivity and activity will be compared with DAWBA ADHD diagnosis to obtain the best predictive model based on QbTest scores that discriminates between ADHD 'positive' and ADHD 'negative' gold standard DAWBA diagnosis.
- Side-effects scale[31]: Side-effects scale will be completed as a control check in medicated participants to ensure greater speed to diagnosis/medication normalisation is not off-set by greater side-effects.
- SNAP-IV[32]: The proportion of patients achieving symptom normalisation assessed via the SNAP-IV. If the young person receives a QbTest on medication (Qb2), the timing on the 3-month SNAP-IV will be moved to coincide with Qb2 to provide a direct comparison of subjective (SNAP-IV) and objective (QbTest) measures. The SNAP-IV is a rating scale designed to assess ADHD symptoms.
- SDQ[30]: The SDQ is a brief behavioural screening questionnaire which can be used as part of a clinical assessment.

For peer review only - http://bmjopen.bmj.com/site/about/guidelines.xhtml

- C-GAS (Children's Global Assessment Scale[33]): Clinician opinion of patient outcome will be assessed via the C-GAS. The C-GAS is a 0-100 scale which that integrates psychological, social and academic functioning in children.
- EQ-5D-Y (EuroQol Five Dimensions Heath Questionnaire-Youth[34]): Child health-related quality of life will be assessed using the EQ-5D-Y.
- A resource collection profile tool will be used. It will encompass elements of a CSRI (Client Service Receipt Inventory[35]) often used in mental health studies but will be a specifically designed economic collection pro-forma for the purpose of this study. It will collect demographic details as well as information on all the services used by the child and family borne costs to be estimated. Indirect costs such as time lost from work incurred by the child's parents or carers will further be recorded. This measure will enable a societal wide perspective for a cost-effectiveness analysis of the QbTest. The DAWBA[29], QbTest[22], SDQ[30,] Side-effects scale[31], , SNAP-IV[32], C-GAS[33], EQ-5D-Y[34] and CSRI[35] all have established reliability, validity and history of use in clinical and research settings.

Feasibility and Acceptability

QbTest opinion questionnaire and interview: Clinician and patient opinion of the QbTest will be assessed via a questionnaire, developed by CLH and currently used to assess QbTest opinion in on-going studies at the Queens Medical Centre, Nottingham. This will provide information on the acceptability of QbTest in routine NHS settings. A sub-sample (n = 20) of families and clinicians will be invited to participate in qualitative interviews to further explore acceptability and feasibility of the QbTest. The sub-sample will be chosen at random from each participating site, using a random number generator.

Table 1 displays a summary of measures, the informant and the time point of completion. All measures will have a one-month window for completion, with the exception of the clinic pro-forma which must be completed during or just after the clinic appointment and the QbTest which must form part of the diagnostic or medication assessment. The collected outcome measures for participants who drop-out the trial before 6-months will be included in analysis,

For peer review only - http://bmjopen.bmj.com/site/about/guidelines.xhtml

no further outcome measures will be collected from these participants after they terminate their participation.

Table 1. Synoptic table of study measures

Measure	Baseline	4-8 weeks after medication initiation*	3 months	6 months
QbTest (C)	x	x		
Pro-forma (C)**	x	x	x	x
C-GAS (C)	x		x	x
DAWBA(P&T)	x			
SDQ (P&T)	x			x
SNAP IV (P&T)	x	x	x	x
EQ-5D-Y (P)	x		x	x
CRSI (P)	x		x	x
QbTest Opinion (C&P)				x
Sub-sample clinician/family interview (n=20)				x

Note. C = Clinician completed. P = Parent/carer completed. T = Teacher completed. *if ADHD diagnosis record and medicated within 3 months of baseline assessment (in place of corresponding 3 month assessment in grey) **Completed at every appointment.

Sample size and justification

The sample size calculation was based on an audit study data from the Department of Community Pediatrics at the Medway NHS Trust (Selby, 2013, unpublished data).

For peer review only

Calculations based on this audit study data showed that the mean number of visits needed to achieve an ADHD diagnosis before introduction of the QbTest (control rate) for children aged 6-14-year olds was 2.94 visits and following the introduction of QbTest a diagnosis was reached in a mean of 2.18 visits. Following consultation with stakeholders, it was agreed that this difference (2.94-2.18) represented the minimum clinically important difference (MCID), with any smaller difference in mean clinic visits being of debatable value. Therefore, 71 patients in each study group will be required to detect a mean count difference of the above magnitude with 80% power at two tailed 0.05 significance level [36 37], assuming the number of visits follows a Poisson distribution.

Given the evidence that the Intraclass Correlation Coefficients (ICC) of mental health measures across GP centers is extremely low[38-40], and results from the Medway audit data indicate that the number of visits needed to achieve an ADHD diagnosis was homogeneous across centers, we will assume that center effects will not influence the sample size calculation for this study. After taking into account a 20% attrition rate, the final total sample size will be 178. The same calculation performed with 90% power would require a total sample of 234 participants. We aim to recruit 178 participants as a minimum and 234 participants as a maximum. Software Stata 13 was used for power analysis.

Randomisation and blinding

Once consent has been obtained from participants, their information will be entered onto a web-based randomisation system (set up by University of Nottingham Clinical Trials Unit; CTU). The arm to which a participant is assigned will be determined by a computer generated pseudo-random code using random permuted blocks of varying size, created by the Nottingham CTU in accordance with their standard operating procedure and held on a secure server. Participants will be allocated with equal probability to each arm (QbO and QbB) with stratification by site. All participants will undergo the same research measures, including the QbTest. It is the time at which the report is made available to the clinician and patient that is randomised (immediately versus 6-months later). Outcome assessors for all measures will be blind to which arm the participant is in. There are no anticipated events
In which participant unblinding would be necessary. There is an allocated un-blinded research team member (CKH) to provide control checks where required.

For peer review only - http://bmjopen.bmj.com/site/about/guidelines.xhtml

Data analysis plan

The analysis will be conducted on an Intention to treat (ITT) basis. Exploratory analysis will be conducted first for both outcome and patient background variables; descriptive statistics of each variable will be presented separately for each group at each follow-up point, with means and standard deviations for normally distributed variables, medians (inter-quartile range) for skewed variables and frequency (percentage) for categorical variables. Missing values will be checked and reported. Multiple imputation will be used to hand missing values, based on a multilevel modelling approach.

To compare the number of visits needed to achieve an ADHD diagnosis (either confirmed or excluded) between groups, Poisson regression with binary group status as the explanatory variable will be implemented. To compare clinician's confidence in their diagnostic decisions, multilevel modelling with patient as a level 2 unit will be used to take into account the non-independence within patient data due to repeated measures[41].

Kappa statistics will be used to reflect the stability of diagnosis between first confirmed diagnosis and diagnosis rerated at 6-month follow up time. Kappa statistics will be reported for each group and the stability of diagnosis will be compared between arms using logistic regression. The same analysis approach will be implemented to explore the stability of diagnosis confidence between time of 1st confirmed diagnosis and 6-month follow-up. To assess the diagnosis accuracy, the sensitivity, specificity, likelihood ratio (LR) ve+, LR ve-, positive predictive value (PPV) and negative predictive value (NPV) will be reported for each group and the test performance will be compared between QbO and QbB arms[42, 43]. Receiver Operating Characteristic (ROC) curve analyses will be used to obtain the best predictive model based on QbTest scores that discriminates between ADHD 'positive' and ADHD 'negative' gold standard DAWBA diagnoses.

For treatment related outcomes (phase 2) outcome measures such as SNAP-IV, side effects scale, SDQ and C-GAS scores, multilevel modelling with patient as a level 2 unit will be again applied to quantify the difference between QbO and QbB arms.

For time to event variables such as time to diagnosis (in days), survival analysis using log-rank test will be performed for group comparison and Kaplan-Meier survival curves will be displayed for each group. Logistic regression will be used to compare the proportion of

For peer review only - http://bmjopen.bmj.com/site/about/guidelines.xhtml

normalisation between two groups at 6-month follow up time. For all regression modelling to explore the difference between arms, group status will be included as explanatory variables. Data transformation would be needed for skewed outcome variables.

Health economic evaluation

Economic evaluation will be completed primarily from a health service perspective but in addition from a societal perspective. A cost effectiveness and cost utility analysis of the treatment options will be conducted. This will include incremental cost effectiveness (ICER) of Qbtest versus usual care; and present cost effectiveness acceptability curves (CEACs) for the diagnostic / treatment options. CEACs enable a probabilistic visual interpretation of the health economic analysis that can be used by decision makers to assist in their choice of health service delivery.

Implementation

To assess feasibility and acceptability we shall look at scores on the QbTest feedback questionnaires. High scores will be taken to indicate high acceptability and feasibility. Mean scores for individual items on each questionnaire will be assessed to determine which aspects of QbTest are perceived negatively or positively by clinicians and service users. Data from clinicians and patients who participate in interviews will be thematically analysed according to the principles of Braun and Clarke[44] to assess themes on the acceptability of QbTest, including patients' opinion on reduced length or number of clinic visits.

Data Monitoring

No interim analysis or analyses for safety or efficacy are planned. Access to data will be restricted to trial team members and associated regulatory authorities as indicated in the sponsor agreement between sites and individual participant information sheets. The Chief Investigator (CH) shall oversee study management, with oversight from the rest of the research team. A sample (10% of the data) will be checked on a regular basis for verification of all entries made. Where corrections are required these will carry a full audit trail and justification, independent from the research team. There are no anticipated adverse effects of the QbTest, all adverse events will be recorded and monitored and the Chief Investigator (CH) will determine seriousness and causality and report the event to the ethics committee.

For peer review only - http://bmjopen.bmj.com/site/about/guidelines.xhtml

The trial is overseen by an independent CLAHRC East Midlands Scientific Committee. The members of the committee are drawn externally from outside the institutions of the research team members and the trial sponsor.

STUDY LIMITATIONS

The diagnosis and management of ADHD is inconsistent, as such the 'assessment as usual' practice will vary across sites. In order to document this difference each site completed a questionnaire prior to their participation in the trial detailing their 'assessment as usual' procedure. Furthermore, basic descriptions of 'assessment as usual' will be recorded in the pro-forma (such as number and length of appointments, decision making and medication). Given this is a pragmatic trial conducted in real-world settings we are interested in the impact of adding QbTest feedback to 'assessment as usual' – without changing other aspects of practice. In order to minimise the trial results being influenced by practice in any one site, we are recruiting participants across multiple sites in different regions of the country and include both CAMHS and community paediatrics. In our design, we have attempted to control for variations between sites by stratification of randomisation by site.

ETHICS AND DISSEMINATION

The study received ethical approval (protocol version 1.2; 05.06.2014) from NRES Committee West Midlands – Coventry and Warwick (14/WM/0166) and has currently received local R&D approval from Alder Hey Children's NHS Foundation Trust, Medway NHS Foundation Trust, Central Manchester University Hospitals NHS Foundation Trust, Sussex Partnership NHS Trust and Lincolnshire Partnership NHS Foundation Trust. Patient recruitment has started at these sites and additional R&D approvals at other sites are in progress. The study is sponsored by the University Of Nottingham; neither the sponsor nor the funders will be involved in the analysis of study data or report writing. QbTech will provide QbTest reports to the study team, which will be analysed by BG, from the University Of Nottingham. Only the research team will have access to the study data, data generated from the trial will be available for inspection by the ethics and R&D committees upon request. Changes to the protocol will be communicated to the ethics committee by the lead research fellow (CLH). The process for obtaining participant informed consent or assent and parent / guardian informed consent will be in accordance with the ethical guidance, and Good Clinical Practice (GCP). The investigator or their nominee and the participant or other legally authorised representative (such as the child's parent) shall both sign and date the informed

For peer review only - http://bmjopen.bmj.com/site/about/guidelines.xhtml

consent forms (Appendix A & B) before the person can participate in the study. Written consent will be required from young people aged 16-years and over and their parents. If the young person is under 16-years of age, parental consent will be required, with the young person's written or verbal assent. Individual participant medical information obtained as a result of this study are considered confidential and disclosure to third parties is prohibited unless warranted by an adverse event. Participant confidentiality will be further ensured by utilising identification code numbers to correspond to treatment data in the computer files. No post-trial care is required.

The primary aim of this study is to determine whether using QbTest in routine NHS settings can accelerate time to correct diagnosis, with a secondary aim of examining whether the QbTest can improve patient outcome. Currently, there are few trials conducted in routine NHS settings with the aim of improving the ADHD care pathway, despite evidence to suggest suboptimal care standards and rising socio-economic burdens. The findings of this study will help to demonstrate whether the QbTest is clinically useful and financially viable in standard care. The findings of the trial will be submitted for publication in appropriate journals regardless of outcome (in accordance with the recommendations of CONSORT) and to members of the public.

Acknowledgements: We would like to thank the site Principle Investigators: Adrian Williams (Alder Hey), Dr. Kim Selby (Medway), Dr. Samina Holsgrove (Central Manchester), Dr. Ify Omeneka (Warrington), Dr. Ann-Marie Skarstam (Sussex, Hastings), Dr. Sarah Curran (Sussex, Maidstone), Dr. Neeta Kulkarni (Leicester), Dr. Julie Clarke (Lincoln), Dr. Maria Moldavsky and Dr. Dilip Nathan (Nottingham) for their support. We would also like to thank: Teresa Jennings, Jo Dodd, Aaron Hobson, Sarah Murphy, Ann-Maria Regan, Susan Good, Gail Melvin, Serena Gregory, Charmaine Kohn and Julie Moss for facilitating the research at their respective sites. Finally, we would like to extend our thanks and appreciation to members of QbTech: Tony Doyle, Charlotte Keizer, Hans Bostrom and Fredrik Ulberstad for their advice and ongoing support.

Contributors: CH is the Chief Investigator and conceived and designed the study with the assistance of KS (co-investigator), DD (co-investigator), BG (medical statistician), and members of Qbtech (Hans Bostrom, Fredrik Ulberstad and Tony Doyle; co-investigators). CLH (lead research fellow) and CH wrote the protocol with approval from all authors. BG

For peer review only - http://bmjopen.bmj.com/site/about/guidelines.xhtml

designed and wrote the statistical analysis plan. CLH, CH, AZV (researcher), GW (research assistant), CKH (clinical trials manager), KS and MJ (co-investigator) contributed to the drafting of the manuscript. All authors critically revised the manuscript for its important intellectual content. All authors read and approved the final manuscript.

Funding: The study is funded as part of the NIHR Collaborations in Leadership in Applied Health Research and Care (CLAHRC) East Midlands, funded by a central grant from the National Institute for Health Research (NIHR), with funding from QbTech in the form of QbTest systems and returning raw data back to the study sites (QbTest reports). The study sponsor and funders have no role in study design, including; collection, management, analysis and interpretation of data; writing of the report; and the decision to submit the report for publication.

Trial Sponsor: University of Nottingham, Mr Paul Cartledge: Kings Meadow Campus, Lenton Lane, Nottingham, NG7 2NR, sponsor@nottingham.ac.uk (REF: 14046).

Competing interests: None.

Ethics approval: This protocol was approved by NRES Committee West Midlands – Coventry and Warwick (14/WM/0166) on 11th July 2014.

Provenance and peer review: Not commissioned; externally peer reviewed.

REFERENCES

1. NICE. Attention deficit hyperactivity disorder: diagnosis and management of ADHD in children, young people and adults. *Clinical Guideline 72*. London: National Institute for Health and Clinical Excellence 2008.
2. Magyary D, Brandt, P. A decision tree and clinical paths for the assessment and management of children with ADHD. *Issues in mental health nursing* 2002; 23.6: 553-566.

For peer review only - http://bmjopen.bmj.com/site/about/guidelines.xhtml

3. Schlander M. Child and Adolescent Psychiatry and Mental Health. *Child and adolescent psychiatry and mental health* 2007;1(13):1-13.

4. King S, Griffin S, Hodges Z, Weatherly H, Asseburg C, Richardson G, et al. A systematic review and economic model of the effectiveness and cost-effectiveness of methylphenidate, dexamfetamine and atomoxetine for the treatment of attention deficit hyperactivity disorder in children and adolescents. *Health Technology Assessment (Winchester, England)* 2006;10(23):iii-iv, xiii-146.

5. D'Amico F, Knapp M, Beecham J, Sandberg S, Taylor E, Sayal K. Use of services and associated costs for young adults with childhood hyperactivity/conduct problems: 20-year follow-up. *The British Journal of Psychiatry* 2014; 204.6: 441-447.

6. Bolea-Alamañac B, Nutt DJ, Adamou M, Asherson P, Bazire S, Coghill D, et al. Evidence-based guidelines for the pharmacological management of attention deficit hyperactivity disorder: Update on recommendations from the British Association for Psychopharmacology. *Journal of Psychopharmacology* 2014;28(3):179-203.

7. Ogundele MO, Ayyash HF, Banerjee S. Role of computerised continuous performance task tests in ADHD. *Progress in Neurology and Psychiatry* 2011;15(3):8-13.

8. Group MC. A 14-month randomized clinical trial of treatment strategies for attention-deficit/hyperactivity disorder. *Archives of General Psychiatry* 1999;56(12):1073.

9. McCarthy S, Cranswick N, Potts L, Taylor E, Wong IC. Mortality associated with attention-deficit hyperactivity disorder (ADHD) drug treatment. *Drug safety* 2009;32(11):1089-96.

10. Sonuga-Barke EJ, Sergeant JA, Nigg J, Willcutt E. Executive dysfunction and delay aversion in attention deficit hyperactivity disorder: nosologic and diagnostic

For peer review only

implications. *Child and adolescent psychiatric clinics of North America* 2008;17(2):367-84.

11. Forbes GB. Clinical utility of the Test of Variables of Attention (TOVA) in the diagnosis of attention-deficit/hyperactivity disorder. *Journal of Clinical Psychology* 1998;54(4):461-76.
12. Sandford J, Turner A. Integrated visual and auditory continuous performance test manual. *Richmond, VA: BrainTrain* 2002.
13. Riccio CA, Cohen MJ, Hynd GW, Keith RW. Validity of the auditory continuous performance test in differentiating central processing auditory disorders with and without ADHD. *Journal of Learning Disabilities* 1996;29(5):561-66.
14. Conners CK, Staff MHS. Conners' Continuous Performance Test II (CPT II V. 5). *North Tonawanda, NY: Multi-Health Systems Inc* 2000.
15. Vogt C, Williams T. Early identification of stimulant treatment responders, partial responders and non-responders using objective measures in children and adolescents with hyperkinetic disorder. *Child and Adolescent Mental Health* 2011;16(3):144-49.
16. Losier BJ, McGrath PJ, Klein RM. Error patterns on the continuous performance test in non-medicated and medicated samples of children with and without ADHD: a meta-analytic review. *Journal of Child Psychology & Psychiatry & Allied Disciplines* 1996;37(8):971-87.
17. Riccio CA, Waldrop JJ, Reynolds CR, Lowe P. Effects of stimulants on the continuous performance test (CPT): implications for CPT use and interpretation. *Journal of Neuropsychiatry & Clinical Neurosciences* 2001;13(3):326-35.
18. Fernandez-Jaen A, Fernandez-Mayoralas DM, Pardos A, Calleja-Perez B, Munoz Jareno N. Clinical and cognitive response to extended-release methylphenidate

For peer review only

(Medikinet) in attention deficit/hyperactivity disorder: efficacy evaluation. *Advances in Therapy* 2009;26(12):1097-110.

19. Huang Y-S, Chao C-C, Wu Y-Y, Chen Y-Y, Chen C-K. Acute effects of methylphenidate on performance during the Test of Variables of Attention in children with attention deficit/hyperactivity disorder. *Psychiatry and Clinical Neurosciences* 2007;61(3):219-25.
20. Solanto M, Newcorn J, Vail L, Gilbert S, Ivanov I, Lara R. Stimulant drug response in the predominantly inattentive and combined subtypes of attention-deficit/hyperactivity disorder. *Journal of Child and Adolescent Psychopharmacology* 2009;19(6):663-71.
21. Ulberstad F. QbTest Technical Manual. *Stockholm, Sweden: Qbtech AB* 2012.
22. Reh V, Schmidt M, Lam L, Schimmelmann BG, Hebebrand J, Rief W, et al. Behavioral Assessment of Core ADHD Symptoms Using the QbTest. *Journal of Attention Disorders* 2013: Doi 1087054712472981.
23. Reh v, Schidt M, Rief W, Christiansen H. Preliminary evidence for altered motion tracking-based hyperactivity in ADHD siblings. *Behavioral and Brain Functions 2014;* 10: DOI:10.1186/1744-9081-10-7.
24. Wehmeier PM, Schacht A, Wolff C, Otto WR, Dittmann RW, Banaschewski T. Neuropsychological outcomes across the day in children with attention-deficit/hyperactivity disorder treated with Atomoxetine: results from a placebo-controlled study using a computer-based continuous performance test combined with an infra-red motion-tracking device. *Journal of Child & Adolescent Psychopharmacology* 2011;21(5):433-44.

For peer review only

25. Ramtvedt BE, Ronias E, Aabech HS, Sundet KS. Clinical gains from including both dextroamphetamine and methylphenidate in stimulant trials. *Journal of child and adolescent psychopharmacology* 2013; 23: 597-604.

26. Günther T, Kahraman-Lanzerath B, Knospe EL, Herpertz-Dahlmann B, Konrad K.Modulation of Attention-Deficit/Hyperactivity Disorder Symptoms by Short-and Long-Acting Methylphenidate over the Course of a Day. *Journal of child and adolescent psychopharmacology* 2012; 22(2): 131-138.

27. Vogt C, Shameli A. Assessments for attention-deficit hyperactivity disorder: Use of objective measurements. *The Psychiatrist* 2011;35(10):380-83.

28. Oades RD, Myint A-M, Dauvermann MR, Schimmelmann BG, SCharz M. Attention-deficit hyperactivity disorder (ADHD) and glial integrity: an exploration of associations of cytokines and kynurenine metabolites with symptoms and attention. *Behavioural and Brain Functions 2010;*6 :32: 1-19.

29. Goodman R, Ford T, Richards H, Gatward R, Meltzer H. The Development and Well-Being Assessment: description and initial validation of an integrated assessment of child and adolescent psychopathology. *Journal of Child Psychology and Psychiatry* 2000;41(05):645-55.

30. Goodman R. The Strengths and Difficulties Questionnaire: a research note. *Journal of Child Psychology and Psychiatry* 1997;38(5):581-86.

31. Hill P, Taylor E. An auditable protocol for treating attention deficit/hyperactivity disorder. *Archives of disease in childhood* 2001;84(5):404-09.

32. Swanson JM, Sandman CA, Deutsch C, Baren M. Methylphenidate hydrochloride given with or before breakfast: I. Behavioral, cognitive, and electrophysiologic effects. *Pediatrics* 1983;72(1):49-55.

For peer review only

33. Shaffer D, Gould MS, Brasic J, Ambrosini P, Fisher P, Bird H, et al. A children's global assessment scale (CGAS). *Archives of General Psychiatry* 1983;40(11):1228-31.

34. Wille N, Badia X, Bonsel G, Burström K, Cavrini G, Devlin N, et al. Development of the EQ-5D-Y: a child-friendly version of the EQ-5D. *Quality of Life Research* 2010;19(6):875-86.

35. Beecham J, Knapp M. *Costing psychiatric interventions*: Gaskell In Measuring Mental Health Needs. Edited by Thornicroft G. London, 2001.

36. Feiveson A. Power by simulation. *Stata J* 2002;2(2):107-24.

37. Van Belle G. *Statistical rules of thumb*: John Wiley & Sons, 2011.

38. Smeeth L, Ng ES-W. Intraclass correlation coefficients for cluster randomized trials in primary care: data from the MRC Trial of the Assessment and Management of Older People in the Community. *Controlled clinical trials* 2002;23(4):409-21.

39. Elley CR, Kerse N, Chondros P, Robinson E. Intraclass correlation coefficients from three cluster randomised controlled trials in primary and residential health care. *Australian and New Zealand journal of public health* 2005;29(5):461-67.

40. Sayal K, Daley D, James M, Yang M, Batty MJ, Taylor JA, et al. Protocol Evaluating the effectiveness of a school-based group programme for parents of children at risk of ADHD: the 'PArents, Teachers and CHildren WORKing Together (PATCHWORK)'cluster RCT protocol. *BMJ open* 2012;2(5). Doi:10.1136/bmjopen-2012-001783

41. Goldstein H. *Multilevel statistical models*: John Wiley & Sons, 2011.

For peer review only

42. Dinnes J, Deeks J, Kirby J, Roderick P. A methodological review of how heterogeneity has been examined in systematic reviews of diagnostic test accuracy. *Health Technology Assessment* 2005;9(12).

43. Mallett S, Halligan S, Thompson M, Collins GS, Altman DG. Interpreting diagnostic accuracy studies for patient care. *BMJ: British Medical Journal* 2012;345.

44. Braun V, Clarke V. Using thematic analysis in psychology. *Qualitative research in psychology* 2006;3(2):77-101.

For peer review only

Protocol investigating the clinical utility of an objective measure of activity and attention (QbTest) on diagnostic and treatment decision-making in children and young people with ADHD: 'Assessing QbTest Utility in ADHD' (AQUA) a randomised controlled trial

Protocol investigating the clinical utility of an objective measure of activity and attention (QbTest) on diagnostic and treatment decision making in children and young people with ADHD: A randomised controlled trial

For peer review only

Authors

[1]Charlotte L. Hall, Research Fellow, CLAHRC, University of Nottingham, Nottingham, UK

[2]Gemma M. Walker, Research Assistant, CLAHRC, University of Nottingham, Nottingham, UK

[3]Althea Z. Valentine, Researcher, CLAHRC, University of Nottingham, Nottingham, UK

[4]Boliang Guo, Medical Statistician, CLAHRC, University of Nottingham, Nottingham, UK

[5]Catherine Kaylor-Hughes, Clinical Trials Manager, CLAHRC, University of Nottingham, Nottingham, UK

[6]Marilyn James, Professor of Health Economics, Division of Rehabilitation and Ageing, University of Nottingham, Nottingham, UK

[7]David Daley, Professor of Psychological Intervention and Behaviour Change, Division of Psychiatry & Applied Psychology, School of Medicine, University of Nottingham, Nottingham, UK

[8]Kapil Sayal, Professor of Child & Adolescent Psychiatry, Developmental Psychiatry, University of Nottingham, Queens Medical Centre, Nottingham, UK

[9]Chris Hollis, Professor of Child & Adolescent Psychiatry, Developmental Psychiatry, University of Nottingham, Queens Medical Centre, Nottingham, UK

Corresponding Author

[9]Chris Hollis,

Institute of Mental Health

University of Nottingham

Triumph Road

Nottingham, NG7 2TU

Chris.hollis@nottingham.ac.uk
0115 8230258

Key words: ADHD, Continuous Performance Test (CPT), QbTest, diagnosis, treatment
Word count: 4738 ~~4321~~ (excluding title page, abstract, summary, references and figures)

ABSTRACT

Introduction

The National Institute for Health and Care Excellence (NICE) guidelines for attention deficit/hyperactivity disorder (ADHD) state that young people need to have access to the best evidence-based care to improve outcome. The current 'gold standard' ADHD diagnostic assessment combines clinical observation with subjective parent, teacher and self-reports. In routine practice, reports from multiple informants may be unavailable or contradictory, leading to diagnostic uncertainty and delay. The addition of objective tests of attention and activity may help reduce diagnostic uncertainty and delays in initiating treatment leading to improved outcomes. This trial aims to investigate whether providing clinicians with an objective report of levels of attention, impulsivity and activity can lead to an earlier, and more accurate, clinical diagnosis and improved patient outcome.

Methods and analysis

This multi-site randomised controlled trial (RCT) will recruit young people (aged 6-17-years old) who have been referred for an ADHD diagnostic assessment at Child and Adolescent Mental Health Services (CAMHS) and Community Paediatric clinics across England. Routine clinical assessment will be augmented by the QbTest, incorporating a computerized continuous performance test (CPT) and infrared motion tracking of activity. The participant will be randomised into one of two study arms: QbOpen (clinician has immediate access to a QbTest report): QbBlind (report is withheld until the study end). Primary outcomes are time to diagnosis and diagnostic accuracy. Secondary outcomes include clinician's diagnostic confidence and routine clinical outcome measures. Cost-effective analysis will be conducted, alongside a qualitative assessment of the feasibility and acceptability of incorporating QbTest in routine practice.

Ethics and dissemination

For peer review only

The findings from the study will inform commissioners, clinicians and managers about the feasibility, acceptability, clinical utility and cost effectiveness of incorporating QbTest into routine diagnostic assessment of young people with ADHD.

The results will be submitted for publication in peer-reviewed journals. The study has received ethical approval.

~~The study has received ethical approval from NRES West Midlands – Coventry and Warwick.~~

Trial registration: NCT02209116

ARTICLE SUMMARY

Article focus

- Multi-site pragmatic randomised controlled trial (RCT) investigating the clinical utility of adding QbTest to the standard assessment and management of attention deficit/hyperactivity disorder (ADHD) in young people in routine NHS settings.

Key messages

- There has been significant increase in the diagnosis and treatment of ADHD in the past 30 years, placing additional demands on the NHS.
- The current 'gold standard' ADHD diagnostic assessment combines clinical observation with subjective parent, teacher and self-reports. However, in routine NHS practice, informant reports are often unavailable or contradictory, leading to diagnostic uncertainty and delay.
- The addition of objective tests of attention and activity may help reduce diagnostic uncertainty and delays in initiating treatment leading to improved outcomes. This study will inform practice by addressing the clinical utility and feasibility of implementing a standardised continuous performance test combined with infra-red motion tracking of activity (QbTest) as part of the diagnostic and management procedure for ADHD.

Strengths and limitations

- A pragmatic diagnostic RCT design in standard NHS settings.
- The focus of the trial is the impact of an objective assessment technology on clinical decision making and patient outcomes.

For peer review only

- There is limited data regarding the clinical utility, cost-effectiveness and acceptability of using the QbTest to aid ADHD assessment and treatment.

INTRODUCTION

Attention Deficit/Hyperactivity Disorder (ADHD) is the most common neuro-developmental disorder and affects 3-5% of children and young people[1]. The core symptoms of ADHD include poor attention, hyper-activity and impulsivity. National Institute for Health and Care Excellence (NICE)[1] guidelines provide a blueprint for the diagnosis and management of ADHD in England and Wales and indicate the need for young people with ADHD to have access to the best evidence-based care in order to fulfil their potential and prevent poor outcome. However, in practice, delivery and quality of care is ad-hoc, with little consistency in assessment, diagnosis or management[2]. ADHD frequently co-exists with other neurodevelopmental and psychiatric disorders and is a risk factor for major educational, social and occupational impairment, placing a huge burden on the NHS, social care and criminal justice systems. There has been a rapid growth in diagnosis over the last 30 years with the number of children recognised and treated for ADHD in the UK increasing almost 10 fold from the early 1980s[1], and spending on medication for ADHD increasing seven fold between 1998 and 2005[3]. The cost of initial specialist assessment for ADHD is estimated at £23 million annually in England and Wales[4], and drug costs for ADHD in England during 2012 was expected to exceed £78 million[3], while indirect costs to families include parental mental ill health, time off work and loss of earnings are even higher[5]. Increasing recognition of ADHD as a lifespan condition is placing a new demand on the NHS to provide diagnostic and treatment services for children, adolescents and adults, exposing serious limitations in existing methods of assessment and management.

There is no single test, or biomarker used to diagnose the disorder[6]. In the absence of any objective measure to identify ADHD, clinical assessment and diagnosis is based on the

For peer review only

clinician's integration of various forms of subjective information including direct observation and reports from parents, teachers and young people. This approach is heavily reliant on subjective measures and clinical interpretation, which can lead to lack of reliability and consistency in the diagnosis of ADHD[7] and furthermore, the process of 'gold standard' clinical interviews and data collection from multiple informants is time consuming and often difficult to conduct in real world settings with frequent missing data and inconsistencies between reports leading to and diagnostic uncertainty and delay.

Additionally, whilst treatments for ADHD are highly efficacious in carefully managed research settings[1], in standard community care the outcome of treatment may be sub-optimal. Aside from delays in initiating treatment caused by diagnostic uncertainty, once on medication, children may not be reviewed sufficiently frequently for clinicians to detect non- or patial-response, or to establish the optimal dose for each child. The U.S. National Institute of Mental Health (NIMH) Multimodal Treatment study of ADHD (MTA) showed that careful medication management can significantly improve outcomes, doubling the normalisation rate from 25% in routine community care to almost 60% when using a strategy of careful dose titration and frequent monitoring of outcome[8]. The NICE[1] ADHD guidelines recommends that during the titration phase, symptoms should be closely monitored using rating scales. However, audit data within the East Midlands showed that community care for ADHD falls well below the standards for titration and monitoring set out in the MTA and NICE guidelines (CLAHRC-NDL, 2013, unpublished audit). A further consequence of sub-optimal treatment response in routine care is poor medication adherence. In the U.K., 50% of patients have stopped ADHD medication after 18 months and 80% after 3 years[9].

Objective Assessment Measures in ADHD

One approach to improving assessment and outcomes in routine care is to add objective laboratory measures of activity and attention for both diagnostic assessment and treatment optimisation[5]. Objective measures have the potential to augment and streamline current practice in order to shorten assessment time, increase diagnostic accuracy, reduce delays in treatment, and optimise treatment response.

Continuous Performance Test

For peer review only - http://bmjopen.bmj.com/site/about/guidelines.xhtml

A Continuous Performance Test (CPT) is a neuropsychological test that measures the individual's capacity to sustain attention (vigilance) and inhibit inappropriate responses (impulsivity), which can be used alongside clinical evaluation to inform the diagnostic process[10]. Typically, a CPT is a computer based programme which involves rapid presentation of visual or auditory stimuli. Participants are asked to respond when a given target occurs but remain passive to non-targets. A standard CPT typically records the child's omission errors (responding when the target is present), commission errors (responding when the target is not present), number of correct responses, reaction time and variability in reaction time. There are several well validated commercially available CPT tests such as the TOVA (Test of Variables of Attention[11]), IVA (Integrated Visual and Auditory[12]), ACPT (Auditory Continuous Performance Test[13]) and the Conner's CPT[14]. These tests are one of the most popular clinic based measures to assess sustained attention in children[16]; with several studies and a meta-analyses showing that children with ADHD perform worse on these tasks than children without ADHD[16]. CPTs have also been shown to be a sensitive measure of medication effects[17]. Several studies have noted improvement in CPT scores in children with ADHD on stimulant medication[15, 18-20]. However, little research has compared CPT scores with more subjective measurements of ADHD[7] and in their recent review Ogundele et al.[7] recommended further research in the use of CPTs compared to standard practice to determine cost effectiveness of these tasks.

A significant limitation of the CPT for the assessment of ADHD is that it doesn't measure the patients' activity levels, which is a core symptom domain of ADHD. Approaches to the objective measurement of activity in ADHD have included wrist-worn actigraphy devices and infra-red motion capture.

QbTest

QbTest (Qbtech Ltd) has been developed to combine a CPT to measure attention and impulsivity with infra-red motion capture of head movement during the CPT to measure activity [21]. The QbTest CPT requires participants to respond to an infrequently presented stimulus (by pressing a button) but ignore all others. Physical activity is measured during the course of the CPT via an infra-red camera that tracks the path of a reflector attached to the participants head (central midpoint). These elements of the test provide information on each

For peer review only

of the three symptom domains of ADHD and provide summary scores for each individual based on deviation from a normative dataset based on age group and gender. There are two versions of the task for children and young people; the task for 6-11-year olds is 15 minutes duration and the task for 12-17-year olds is 20 minutes duration. The QbTest result is complemented by a clinical evaluation and behavioural observation of events that may affect test performance. The QbTest is not a stand-alone diagnostic tool, but has been approved by the U.S. Food and Drug Administration (FDA; Ref: K133382) to supplement standard clinical assessment and treatment follow-up by reducing reliance on measures such as subjective observer reports (which can be biased, incomplete or missing) and augment clinical decision-making.

The research available about the QbTest has shown favourable psychometric characteristics with child participants. For example, Qbtech report data that establishes 85% sensitivity of classification and 92% specificity, and good test-retest scores, which is not influenced by experience with computers (Ulberstad, 2012, unpublished data). Reh et al.[22] investigated the factor structure and concurrent and discriminant validity of QbTest and found the hyperactivity factor correlated with teacher ratings of hyperactive behaviour, providing evidence for the utility of including this additional measure of activity in a CPT. In addition, Reh et al.[23] found the hyperactivity factor could identify intermediate levels of impairment in ADHD siblings, suggesting this factor maybe particularly sensitivity as an intermediate phenotype for ADHD. Their findings also provide initial evidence for the concurrent validity of the three factors (attention, impulsivity, activity), although the authors highlight the need for further research to investigate validity. Wehmeier et al.[24] found QbTest to be a valid measure of treatment outcome and highly correlated with blinded observer ratings of behaviour in placebo-controlled RCTs. QbTest is effective in evaluating ADHD medication effects in children[25 26], and can identify early non-responders[15]. One clinical study found QbTest improved clinical accuracy by reducing the risk of unidentified ADHD when patients were re-evaluated one-year after their initial assessment[27] and another indicated the ability for QbTest to differentiate ADHD from normative controls[28]. Initial audit data (Selby, 2013, unpublished data) suggest that implementation of QbTest in routine ADHD clinics can reduce the time to diagnosis by 30%. This equates to a reduction from an average of three to two out-patient appointments per patient in order to either confirm or exclude a diagnosis of ADHD. These findings indicate potential for QbTest to support the diagnostic assessment and management of ADHD within routine clinical practice; however, there has been no

randomised controlled trial (RCT) to investigate the added clinical value (clinical utility) and economic cost effectiveness of adding QbTest to standard ADHD care pathways within the NHS.

The primary aim of the Assessing QbTest Utility in ADHD-trial (AQUA-Trial) is to determine whether using QbTest in routine NHS settings can accelerate diagnosis without compromising diagnostic accuracy. Secondly, the study aims to examine whether QbTest improves the medication titration process by increasing the proportion of patients normalised after 6 months post baseline assessment and improves patient outcome. The study will also use qualitative methods to explore the barriers, drivers and facilitators to the adoption of the QbTest in routine practice The cost-effectiveness of implementing the QbTest in practice will also be investigated.~~and assess its cost effectiveness~~. The findings will indicate whether establishing QbTest as part of standard practice in ADHD assessment and management is clinically useful, financially viable and acceptable for clinicians and patients.

METHODS AND ANALYSIS

Trial Design

This study is a parallel group single-blind multi-center RCT, exploring feasibility and acceptability, using quantitative and qualitative and health economic evaluations. The study consists of two arms:

1) QbOpen (QbO)

In this arm of the trial participants will complete the QbTest and clinicians, participants and their families will have immediate access to a QbTest report.

2) QbBlind: (QbB):

In this arm of the trial participants will complete the QbTest but the QbTest report will be withheld from the clinician, participant and patient's family until the last outcome measure is completed at 6 months. All participants will receive the same intervention. Specifically, this will be assessment as usual plus a QbTest as part of the diagnostic assessment.

For peer review only

The patients usual care team will be responsible for conducting the QbTest in clinic appointments. The QbTest will be only be conducted by trained QbTest clinicians.

Setting

Child and Adolescent Mental Health Services (CAMHS) and Community Paediatric clinics across 9 different NHS Trusts in England, including Medway NHS Foundation Trust, Alder Hey Children's NHS Foundation Trust, Nottinghamshire Healthcare NHS Trust, Leicestershire Partnership NHS Trust, Sussex Partnership NHS Foundation Trust, United Lincolnshire Hospital NHS Trust, Central Manchester University Hospitals NHS Foundation Trust, Bridgewater Community Healthcare NHS Trust, Nottingham University Hospitals NHS Trust. Additional NHS Trusts may be recruited to meet target recruitment figures.

Recruitment and eligibility

New referrals for a diagnostic evaluation for suspected ADHD will be invited to participate in the research based on the following criteria:

Inclusion criteria

- Age 6-17-years old (at the time of consent)
- Referred to CAMHS or Community Paediatrics for an ADHD diagnostic assessment
- Capable of providing written informed consent (over 16-years old)
- Parental consent (under 16-years old)

Exclusion criteria

- Severe learning disability (to be assessed by clinical judgment)
- Non-fluent English speaking
- Previous or current confirmed diagnosis of ADHD
- Currently receiving ADHD medication

Written information about the trial will be sent to families through the clinic administrators prior to their first appointment. Clinic invitations will be updated and recorded on a password protected database on a weekly basis. Parents and young people who wish to participate will be asked to complete and return a consent form before this appointment. Alternatively,

For peer review only

participants will be consented into the study by the clinical team, Clinical Studies Officer or a member of the research team at their first appointment. Each site will be informed of the monthly recruitment target required in order to meet the study sample size and updated on their monthly progress.

Trial Phases

There are two phases to this study (see Figure 1).

Phase 1, Assessment: The first phase investigates the use of QbTest as a tool to aid diagnosis. Participants will undergo ADHD 'assessment as usual', which varies between clinics, clinicians and cases, but will typically involve an interview with the parent/young person and collection of questionnaires from the parent/young person and teacher. Whilst attending the clinic, the participant will be asked to complete the QbTest at some point during the diagnostic process. Participants will also be asked to complete baseline outcome measures (see measures section).

Phase 2, Treatment: Patients who receive a clinic diagnosis of ADHD and are allocated by clinicians to receive ADHD medication initiated within 3 months of their baseline assessment will be asked to complete a second QbTest (Qb2) 4-8 weeks after medication initiation. This timeframe was chosen to ensure that all participants can complete their second QbTest before the 6-month follow-up.

All participants will stay in the trial for 6-months and will be asked to complete outcome measures at 3 and 6-month follow up, regardless of their diagnosis or whether they receive medication. With the aim of promoting participant retention and completion of follow-up measures, participants will be compensated for their time with a £15 high-street voucher if they remain in the trial until 6-months.

Measures

Blinded members of the research team (CLH, GMW, AVZ,) will be fully trained in all trial assessments and responsible for monitoring the distribution, completion and collection of all outcome measures.

Primary Outcome

The primary outcome is the number of consultations until a confirmed clinical diagnosis is reached, as recorded on a short pro-forma. The pro-forma will be completed by clinicians

For peer review only

after each consultation with the young person and/or family and documents information about appointment duration, diagnosis and medication/treatment. The pro-forma can be provided by contacting the corresponding author.

Secondary Outcomes

The secondary outcomes obtained from the pro-forma are:

- Number of days and duration of visits (in minutes) until a confirmed diagnosis is reached.
- Clinical confidence in diagnostic decision. Clinicians will be required to rate the confidence of their decision on a 7-point Likert scale (Definitely ADHD-Definitely not ADHD).
- Stability in diagnosis. Clinicians will be required to re-rate their diagnostic decision and confidence at 6-months.

Other measures

- Development and Well-being Assessment (DAWBA[29]): The DAWBA is a semi-structured, investigator-based diagnostic interview for child mental health problems, including ADHD, which includes the (Strengths and Difficulties Questionnaire; SDQ[30]) as an initial screen. The parent and teacher DAWBA will be completed to compare the accuracy of clinic diagnosis (in both QbO and QbB arms of the trial) to that of an independent clinical consensus diagnosis made using the DAWBA. Two experienced clinicians, blind to allocation, will review the DAWBA and arrive at a clinical consensus diagnosis.
- QbTest scores: Q-scores for attention, impulsivity and activity will be compared with DAWBA ADHD diagnosis to obtain the best predictive model based on QbTest scores that discriminates between ADHD 'positive' and ADHD 'negative' gold standard DAWBA diagnosis.
- Side-effects scale[31]: Side-effects scale will be completed as a control check in medicated participants to ensure greater speed to diagnosis/medication normalisation is not off-set by greater side-effects.
- SNAP-IV[32]: The proportion of patients achieving symptom normalisation assessed via the SNAP-IV. If the young person receives a QbTest on medication (Qb2), the timing

For peer review only - http://bmjopen.bmj.com/site/about/guidelines.xhtml

on the 3-month SNAP-IV will be moved to coincide with Qb2 to provide a direct comparison of subjective (SNAP-IV) and objective (QbTest) measures. The SNAP-IV is a rating scale designed to assess ADHD symptoms.

- SDQ[30]: The SDQ is a brief behavioural screening questionnaire which can be used as part of a clinical assessment.
- C-GAS (Children's Global Assessment Scale[33]): Clinician opinion of patient outcome will be assessed via the C-GAS. The C-GAS is a 0-100 scale which that integrates psychological, social and academic functioning in children.
- EQ-5D-Y (EuroQol Five Dimensions Heath Questionnaire-Youth[34]): Child health-related quality of life will be assessed using the EQ-5D-Y.
- A resource collection profile tool will be used. It will encompass elements of a CSRI (Client Service Receipt Inventory[35]) often used in mental health studies but will be a specifically designed economic collection pro-forma for the purpose of this study. It will collect demographic details as well as information on all the services used by the child and family borne costs to be estimated. Indirect costs such as time lost from work incurred by the child's parents or carers will further be recorded. This measure will enable a societal wide perspective for a cost-effectiveness analysis of the QbTest. The DAWBA[29], QbTest[22], SDQ[30,] Side-effects scale[31], , SNAP-IV[32], C-GAS[33], EQ-5D-Y[34] and CSRI[35] all have established reliability, validity and history of use in clinical and research settings.

Feasibility and Acceptability

QbTest opinion questionnaire and interview: Clinician and patient opinion of the QbTest will be assessed via a questionnaire, developed by CLH and currently used to assess QbTest opinion in on-going studies at the Queens Medical Centre, Nottingham. This will provide information on the acceptability of QbTest in routine NHS settings. A sub-sample (*n* = 20) of families and clinicians will be invited to participate in qualitative interviews to further explore acceptability and feasibility of the QbTest. The sub-sample will be chosen at random from each participating site, using a random number generator.

For peer review only

Table 1 displays a summary of measures, the informant and the time point of completion. All measures will have a one-month window for completion, with the exception of the clinic pro-forma which must be completed during or just after the clinic appointment and the QbTest which must form part of the diagnostic or medication assessment. The collected outcome measures for participants who drop-out the trial before 6-months will be included in analysis, no further outcome measures will be collected from these participants after they terminate their participation.

Table 1. Synoptic table of study measures

Measure	Baseline	4-8 weeks after medication initiation*	3 months	6 months
QbTest (C)	x	x		
Pro-forma (C)**	x	x	x	x
C-GAS (C)	x		x	x
DAWBA(P&T)	x			
SDQ (P&T)	x			x
SNAP IV (P&T)	x	x	x	x
EQ-5D-Y (P)	x		x	x
CRSI (P)	x		x	x
QbTest Opinion (C&P)				x
Sub-sample clinician/family interview (n=20)				x

For peer review only

Note. C = Clinician completed. P = Parent/carer completed. T = Teacher completed. *if ADHD diagnosis record and medicated within 3 months of baseline assessment (in place of corresponding 3 month assessment in grey) **Completed at every appointment.

Sample size and justification

The sample size calculation was based on an audit study data from the Department of Community Pediatrics at the Medway NHS Trust (Selby, 2013, unpublished data). Calculations based on this audit study data showed that the mean number of visits needed to achieve an ADHD diagnosis before introduction of the QbTest (control rate) for children aged 6-14-year olds was 2.94 visits and following the introduction of QbTest a diagnosis was reached in a mean of 2.18 visits. Following consultation with stakeholders, it was agreed that this difference (2.94-2.18) represented the minimum clinically important difference (MCID), with any smaller difference in mean clinic visits being of debatable value. Therefore, 71 patients in each study group will be required to detect a mean count difference of the above magnitude with 80% power at two tailed 0.05 significance level [36 37],

assuming the number of visits follows a Poisson distribution.

~~assuming the count of visit follows a Poisson distribution. Assuming the number of visits follows a Poisson distribution.~~

Given the evidence that the Intraclass Correlation Coefficients (ICC) of mental health measures across GP centers is extremely low[38-40], and results from the Medway audit data indicate that the number of visits needed to achieve an ADHD diagnosis was homogeneous across centers, we will assume that center effects will not influence the sample size calculation for this study. After taking into account a 20% attrition rate, the final total sample size will be 178. The same calculation performed with 90% power would require a total sample of 234 participants. We aim to recruit 178 participants as a minimum and 234 participants as a maximum. Software Stata 13 was used for power analysis.

Randomisation and blinding

Once consent has been obtained from participants, their information will be entered onto a web-based randomisation system (set up by University of Nottingham Clinical Trials Unit; CTU). The arm to which a participant is assigned will be determined by a computer generated pseudo-random code using random permuted blocks of varying size, created by the Nottingham CTU in accordance with their standard operating procedure and held on a secure server. Participants will be allocated with equal probability to each arm (QbO and QbB) with stratification by site. All participants will undergo the same research measures, including the

For peer review only

QbTest. It is the time at which the report is made available to the clinician and patient that is randomised (immediately versus 6-months later). Outcome assessors for all measures will be blind to which arm the participant is in. There are no anticipated events
In which participant unblinding would be necessary. There is an allocated un-blinded research team member (CKH) to provide control checks where required.

Data analysis plan

The analysis will be conducted on an Intention to treat (ITT) basis. Exploratory analysis will be conducted first for both outcome and patient background variables; descriptive statistics of each variable will be presented separately for each group at each follow-up point, with means and standard deviations for normally distributed variables, medians (inter-quartile range) for skewed variables and frequency (percentage) for categorical variables. Missing values will be checked and reported. Multiple imputation will be used to hand missing values, based on a multilevel modelling approach.
To compare the number of visits needed to achieve an ADHD diagnosis (either confirmed or excluded) between groups, Poisson regression with binary group status as the explanatory variable will be implemented. To compare clinician's confidence in their diagnostic decisions, multilevel modelling with patient as a level 2 unit will be used to take into account the non-independence within patient data due to repeated measures[41].

Kappa statistics will be used to reflect the stability of diagnosis between first confirmed diagnosis and diagnosis rerated at 6-month follow up time. Kappa statistics will be reported for each group and the stability of diagnosis will be compared between arms using logistic regression. The same analysis approach will be implemented to explore the stability of diagnosis confidence between time of 1st confirmed diagnosis and 6-month follow-up. To assess the diagnosis accuracy, the sensitivity, specificity, likelihood ratio (LR) ve+, LR ve-, positive predictive value (PPV) and negative predictive value (NPV) will be reported for each group and the test performance will be compared between QbO and QbB arms[42, 43]. Receiver Operating Characteristic (ROC) curve analyses will be used to obtain the best predictive model based on QbTest scores that discriminates between ADHD 'positive' and ADHD 'negative' gold standard DAWBA diagnoses.

For peer review only - http://bmjopen.bmj.com/site/about/guidelines.xhtml

For treatment related outcomes (phase 2) outcome measures such as SNAP-IV, side effects scale, SDQ and C-GAS scores, multilevel modelling with patient as a level 2 unit will be again applied to quantify the difference between QbO and QbB arms.

For time to event variables such as time to diagnosis (in days), survival analysis using log-rank test will be performed for group comparison and Kaplan-Meier survival curves will be displayed for each group. Logistic regression will be used to compare the proportion of normalisation between two groups at 6-month follow up time. For all regression modelling to explore the difference between arms, group status will be included as explanatory variables. Data transformation would be needed for skewed outcome variables.

Health economic evaluation

Economic evaluation will be completed primarily from a health service perspective but in addition from a societal perspective. A cost effectiveness and cost utility analysis of the treatment options will be conducted. This will include incremental cost effectiveness (ICER) of Qbtest versus usual care; and present cost effectiveness acceptability curves (CEACs) for the diagnostic / treatment options. CEACs enable a probabilistic visual interpretation of the health economic analysis that can be used by decision makers to assist in their choice of health service delivery.

Implementation

To assess feasibility and acceptability we shall look at scores on the QbTest feedback questionnaires. High scores will be taken to indicate high acceptability and feasibility. Mean scores for individual items on each questionnaire will be assessed to determine which aspects of QbTest are perceived negatively or positively by clinicians and service users. Data from clinicians and patients who participate in interviews will be thematically analysed according to the principles of Braun and Clarke[44] to assess themes on the acceptability of QbTest, including patients' opinion on reduced length or number of clinic visits.

Data Monitoring

No interim analysis or analyses for safety or efficacy are planned.. Access to data will be restricted to trial team members and associated regulatory authorities as indicated in the sponsor agreement between sites and individual participant information sheets. The Chief Investigator (CH) shall oversee study management, with oversight from the rest of the

For peer review only - http://bmjopen.bmj.com/site/about/guidelines.xhtml

research team. A sample (10% of the data) will be checked on a regular basis for verification of all entries made. Where corrections are required these will carry a full audit trail and justification, independent from the research team. There are no anticipated adverse effects of the QbTest, all adverse events will be recorded and monitored and the Chief Investigator (CH) will determine seriousness and causality and report the event to the ethics committee. The trial is overseen by an independent CLAHRC East Midlands Scientific Committee. The members of the committee are drawn externally from outside the institutions of the research team members and the trial sponsor.

STUDY LIMITATIONS

The diagnosis and management of ADHD is inconsistent, as such the 'assessment as usual' practice will vary across sites. In order to document this difference each site completed a questionnaire prior to their participation in the trial detailing their 'assessment as usual' procedure. Furthermore, basic descriptions of 'assessment as usual' will be recorded in the pro-forma (such as number and length of appointments, decision making and medication). Given this is a pragmatic trial conducted in real-world settings we are interested in the impact of adding QbTest feedback to 'assessment as usual' – without changing other aspects of practice. In order to minimise the trial results being influenced by practice in any one site, we are recruiting participants across multiple sites in different regions of the country and include both CAMHS and community paediatrics. In our design, we have attempted to control for variations between sites by stratification of randomisation by site.

ETHICS AND DISSEMINATION

The study received ethical approval (protocol version 1.2; 05.06.2014) from NRES Committee West Midlands – Coventry and Warwick (14/WM/0166) and has currently received local R&D approval from Alder Hey Children's NHS Foundation Trust, Medway NHS Foundation Trust, Central Manchester University Hospitals NHS Foundation Trust, ~~and~~ Sussex Partnership NHS Trust and Lincolnshire Partnership NHS Foundation Trust. Patient recruitment has started at these sites and additional R&D approvals at other sites are in progress. The study is sponsored by the University Of Nottingham; neither the sponsor nor the funders will be involved in the analysis of study data or report writing. QbTech will provide QbTest reports to the study team, which will be analysed by BG, from the University Of Nottingham. Only the research team will have access to the study data, data generated from the trial will be available for inspection by the ethics and R&D committees upon

For peer review only

request. Changes to the protocol will be communicated to the ethics committee by the lead research fellow (CLH). The process for obtaining participant informed consent or assent and parent / guardian informed consent will be in accordance with the ethical guidance, and Good Clinical Practice (GCP). The investigator or their nominee and the participant or other legally authorised representative (such as the child's parent) shall both sign and date the informed consent forms (Appendix A & B) before the person can participate in the study. Written consent will be required from young people aged 16-years and over and their parents. If the young person is under 16-years of age, parental consent will be required, with the young person's written or verbal assent. Individual participant medical information obtained as a result of this study are considered confidential and disclosure to third parties is prohibited unless warranted by an adverse event. Participant confidentiality will be further ensured by utilising identification code numbers to correspond to treatment data in the computer files. No post-trial care is required.

The primary aim of this study is to determine whether using QbTest in routine NHS settings can accelerate time to correct diagnosis, with a secondary aim of examining whether the QbTest can improve patient outcome. Currently, there are few trials conducted in routine NHS settings with the aim of improving the ADHD care pathway, despite evidence to suggest suboptimal care standards and rising socio-economic burdens. The findings of this study will help to demonstrate whether the QbTest is clinically useful and financially viable in standard care. The findings of the trial will be submitted for publication in appropriate journals regardless of outcome (in accordance with the recommendations of CONSORT) and to members of the public.

Acknowledgements: We would like to thank the site Principle Investigators: Adrian Williams (Alder Hey), Dr. Kim Selby (Medway), Dr. Samina Holsgrove (Central Manchester), Dr. Ify Omeneka (Warrington), Dr. Ann-Marie Skarstam (Sussex, Hastings), Dr. Sarah Curran (Sussex, Maidstone), Dr. Neeta Kulkarni (Leicester), Dr. Julie Clarke (Lincoln), Dr. Maria Moldavsky and Dr. Dilip Nathan (Nottingham) for their support. We would also like to thank: Teresa Jennings, Jo Dodd, Aaron Hobson, Sarah Murphy, Ann-Maria Regan, Susan Good, Gail ~~Melvin~~ Melvin, Serena Gregory, Charmaine Kohn ,and Julie Moss for facilitating the research at their respective sites. Finally, we would like to extend

For peer review only

our thanks and appreciation to members of QbTech: Tony Doyle, Charlotte Keizer, Hans Bostrom and Fredrik Ulberstad for their advice and ongoing support.

Contributors: CH is the Chief Investigator and conceived and designed the study with the assistance of KS (co-investigator), DD (co-investigator), BG (medical statistician), and members of Qbtech (Hans Bostrom, Fredrik Ulberstad and Tony Doyle; co-investigators). CLH (lead research fellow) and CH wrote the protocol with approval from all authors. BG designed and wrote the statistical analysis plan. CLH, CH, AZV (researcher), GW (research assistant), CKH (clinical trials manager), KS and MJ (co-investigator) contributed to the drafting of the manuscript. All authors critically revised the manuscript for its important intellectual content. All authors read and approved the final manuscript.

Funding: The study is funded as part of the NIHR Collaborations in Leadership in Applied Health Research and Care (CLAHRC) East Midlands, funded by a central grant from the National Institute for Health Research (NIHR), with funding from QbTech in the form of QbTest systems and returning raw data back to the study sites (QbTest reports). The study sponsor and funders have no role in study design, including; collection, management, analysis and interpretation of data; writing of the report; and the decision to submit the report for publication.

Trial Sponsor: University of Nottingham, Mr Paul Cartledge: Kings Meadow Campus, Lenton Lane, Nottingham, NG7 2NR, sponsor@nottingham.ac.uk (REF: 14046).

Competing interests: None.

Ethics approval: This protocol was approved by NRES Committee West Midlands – Coventry and Warwick (14/WM/0166) on 11th July 2014.

Provenance and peer review: Not commissioned; externally peer reviewed.

REFERENCES

For peer review only

1. NICE. Attention deficit hyperactivity disorder: diagnosis and management of ADHD in children, young people and adults. *Clinical Guideline 72*. London: National Institute for Health and Clinical Excellence 2008.

2. Magyary D, Brandt, P. A decision tree and clinical paths for the assessment and management of children with ADHD. *Issues in mental health nursing* 2002; 23.6: 553-566.

3. Schlander M. Child and Adolescent Psychiatry and Mental Health. *Child and adolescent psychiatry and mental health* 2007;1(13):1-13.

4. King S, Griffin S, Hodges Z, Weatherly H, Asseburg C, Richardson G, et al. A systematic review and economic model of the effectiveness and cost-effectiveness of methylphenidate, dexamfetamine and atomoxetine for the treatment of attention deficit hyperactivity disorder in children and adolescents. *Health Technology Assessment (Winchester, England)* 2006;10(23):iii-iv, xiii-146.

5. D'Amico F, Knapp M, Beecham J, Sandberg S, Taylor E, Sayal K. Use of services and associated costs for young adults with childhood hyperactivity/conduct problems: 20-year follow-up. *The British Journal of Psychiatry* 2014; 204.6: 441-447.

6. Bolea-Alamañac B, Nutt DJ, Adamou M, Asherson P, Bazire S, Coghill D, et al. Evidence-based guidelines for the pharmacological management of attention deficit hyperactivity disorder: Update on recommendations from the British Association for Psychopharmacology. *Journal of Psychopharmacology* 2014;28(3):179-203.

7. Ogundele MO, Ayyash HF, Banerjee S. Role of computerised continuous performance task tests in ADHD. *Progress in Neurology and Psychiatry* 2011;15(3):8-13.

For peer review only

8. Group MC. A 14-month randomized clinical trial of treatment strategies for attention-deficit/hyperactivity disorder. *Archives of General Psychiatry* 1999;56(12):1073.

9. McCarthy S, Cranswick N, Potts L, Taylor E, Wong IC. Mortality associated with attention-deficit hyperactivity disorder (ADHD) drug treatment. *Drug safety* 2009;32(11):1089-96.

10. Sonuga-Barke EJ, Sergeant JA, Nigg J, Willcutt E. Executive dysfunction and delay aversion in attention deficit hyperactivity disorder: nosologic and diagnostic implications. *Child and adolescent psychiatric clinics of North America* 2008;17(2):367-84.

11. Forbes GB. Clinical utility of the Test of Variables of Attention (TOVA) in the diagnosis of attention-deficit/hyperactivity disorder. *Journal of Clinical Psychology* 1998;54(4):461-76.

12. Sandford J, Turner A. Integrated visual and auditory continuous performance test manual. *Richmond, VA: BrainTrain* 2002.

13. Riccio CA, Cohen MJ, Hynd GW, Keith RW. Validity of the auditory continuous performance test in differentiating central processing auditory disorders with and without ADHD. *Journal of Learning Disabilities* 1996;29(5):561-66.

14. Conners CK, Staff MHS. Conners' Continuous Performance Test II (CPT II V. 5). *North Tonawanda, NY: Multi-Health Systems Inc* 2000.

15. Vogt C, Williams T. Early identification of stimulant treatment responders, partial responders and non-responders using objective measures in children and adolescents with hyperkinetic disorder. *Child and Adolescent Mental Health* 2011;16(3):144-49.

16. Losier BJ, McGrath PJ, Klein RM. Error patterns on the continuous performance test in non-medicated and medicated samples of children with and without ADHD: a

For peer review only

meta-analytic review. *Journal of Child Psychology & Psychiatry & Allied Disciplines* 1996;37(8):971-87.

17. Riccio CA, Waldrop JJ, Reynolds CR, Lowe P. Effects of stimulants on the continuous performance test (CPT): implications for CPT use and interpretation. *Journal of Neuropsychiatry & Clinical Neurosciences* 2001;13(3):326-35.
18. Fernandez-Jaen A, Fernandez-Mayoralas DM, Pardos A, Calleja-Perez B, Munoz Jareno N. Clinical and cognitive response to extended-release methylphenidate (Medikinet) in attention deficit/hyperactivity disorder: efficacy evaluation. *Advances in Therapy* 2009;26(12):1097-110.
19. Huang Y-S, Chao C-C, Wu Y-Y, Chen Y-Y, Chen C-K. Acute effects of methylphenidate on performance during the Test of Variables of Attention in children with attention deficit/hyperactivity disorder. *Psychiatry and Clinical Neurosciences* 2007;61(3):219-25.
20. Solanto M, Newcorn J, Vail L, Gilbert S, Ivanov I, Lara R. Stimulant drug response in the predominantly inattentive and combined subtypes of attention-deficit/hyperactivity disorder. *Journal of Child and Adolescent Psychopharmacology* 2009;19(6):663-71.
21. Ulberstad F. QbTest Technical Manual. *Stockholm, Sweden: Qbtech AB* 2012.
22. Reh V, Schmidt M, Lam L, Schimmelmann BG, Hebebrand J, Rief W, et al. Behavioral Assessment of Core ADHD Symptoms Using the QbTest. *Journal of Attention Disorders* 2013: Doi 1087054712472981.
23. Reh v, Schidt M, Rief W, Christiansen H. Preliminary evidence for altered motion tracking-based hyperactivity in ADHD siblings. *Behavioral and Brain Functions 2014;* 10: DOI:10.1186/1744-9081-10-7.

For peer review only

24. Wehmeier PM, Schacht A, Wolff C, Otto WR, Dittmann RW, Banaschewski T. Neuropsychological outcomes across the day in children with attention-deficit/hyperactivity disorder treated with Atomoxetine: results from a placebo-controlled study using a computer-based continuous performance test combined with an infra-red motion-tracking device. *Journal of Child & Adolescent Psychopharmacology* 2011;21(5):433-44.

25. Ramtvedt BE, Ronias E, Aabech HS, Sundet KS. Clinical gains from including both dextroamphetamine and methylphenidate in stimulant trials. *Journal of child and adolescent psychopharmacology* 2013; 23: 597-604.

26. Günther T, Kahraman-Lanzerath B, Knospe EL, Herpertz-Dahlmann B, Konrad K.Modulation of Attention-Deficit/Hyperactivity Disorder Symptoms by Short-and Long-Acting Methylphenidate over the Course of a Day. *Journal of child and adolescent psychopharmacology* 2012; 22(2): 131-138.

27. Vogt C, Shameli A. Assessments for attention-deficit hyperactivity disorder: Use of objective measurements. *The Psychiatrist* 2011;35(10):380-83.

28. Oades RD, Myint A-M, Dauvermann MR, Schimmelmann BG, SCharz M. Attention-deficit hyperactivity disorder (ADHD) and glial integrity: an exploration of associations of cytokines and kynurenine metabolites with symptoms and attention. *Behavioural and Brain Functions 2010;*6 :32: 1-19.

29. Goodman R, Ford T, Richards H, Gatward R, Meltzer H. The Development and Well-Being Assessment: description and initial validation of an integrated assessment of child and adolescent psychopathology. *Journal of Child Psychology and Psychiatry* 2000;41(05):645-55.

30. Goodman R. The Strengths and Difficulties Questionnaire: a research note. *Journal of Child Psychology and Psychiatry* 1997;38(5):581-86.

For peer review only

31. Hill P, Taylor E. An auditable protocol for treating attention deficit/hyperactivity disorder. *Archives of disease in childhood* 2001;84(5):404-09.

32. Swanson JM, Sandman CA, Deutsch C, Baren M. Methylphenidate hydrochloride given with or before breakfast: I. Behavioral, cognitive, and electrophysiologic effects. *Pediatrics* 1983;72(1):49-55.

33. Shaffer D, Gould MS, Brasic J, Ambrosini P, Fisher P, Bird H, et al. A children's global assessment scale (CGAS). *Archives of General Psychiatry* 1983;40(11):1228-31.

34. Wille N, Badia X, Bonsel G, Burström K, Cavrini G, Devlin N, et al. Development of the EQ-5D-Y: a child-friendly version of the EQ-5D. *Quality of Life Research* 2010;19(6):875-86.

35. Beecham J, Knapp M. *Costing psychiatric interventions*: Gaskell In Measuring Mental Health Needs. Edited by Thornicroft G. London, 2001.

36. Feiveson A. Power by simulation. *Stata J* 2002;2(2):107-24.

37. Van Belle G. *Statistical rules of thumb*: John Wiley & Sons, 2011.

38. Smeeth L, Ng ES-W. Intraclass correlation coefficients for cluster randomized trials in primary care: data from the MRC Trial of the Assessment and Management of Older People in the Community. *Controlled clinical trials* 2002;23(4):409-21.

39. Elley CR, Kerse N, Chondros P, Robinson E. Intraclass correlation coefficients from three cluster randomised controlled trials in primary and residential health care. *Australian and New Zealand journal of public health* 2005;29(5):461-67.

40. Sayal K, Daley D, James M, Yang M, Batty MJ, Taylor JA, et al. Protocol Evaluating the effectiveness of a school-based group programme for parents of children at risk of ADHD: the 'PArents, Teachers and CHildren WORKing Together

For peer review only

For peer review only - http://bmjopen.bmj.com/site/about/guidelines.xhtml

(PATCHWORK)'cluster RCT protocol. *BMJ open* 2012;2(5). Doi:10.1136/bmjopen-2012-001783

41. Goldstein H. *Multilevel statistical models*: John Wiley & Sons, 2011.

42. Dinnes J, Deeks J, Kirby J, Roderick P. A methodological review of how heterogeneity has been examined in systematic reviews of diagnostic test accuracy. *Health Technology Assessment* 2005;9(12).

43. Mallett S, Halligan S, Thompson M, Collins GS, Altman DG. Interpreting diagnostic accuracy studies for patient care. *BMJ: British Medical Journal* 2012;345.

44. Braun V, Clarke V. Using thematic analysis in psychology. *Qualitative research in psychology* 2006;3(2):77-101.

For peer review only

STANDARD PROTOCOL ITEMS: RECOMMENDATIONS FOR INTERVENTIONAL TRIALS

SPIRIT 2013 Checklist: Recommended items to address in a clinical trial protocol and related documents*

Section/item	Item No	Description
**Administrative information**		
√ Title	1	Descriptive title identifying the study design, population, interventions, and, if applicable, trial acronym
√ Trial registration	2a	Trial identifier and registry name. If not yet registered, name of intended registry
	2b	All items from the World Health Organization Trial Registration Data Set
√ Protocol version	3	Date and version identifier
√ Funding	4	Sources and types of financial, material, and other support
√ Roles and responsibilities	5a	Names, affiliations, and roles of protocol contributors
	5b	Name and contact information for the trial sponsor
	5c	Role of study sponsor and funders, if any, in study design; collection, management, analysis, and interpretation of data; writing of the report; and the decision to submit the report for publication, including whether they will have ultimate authority over any of these activities
	5d	Composition, roles, and responsibilities of the coordinating centre, steering committee, endpoint adjudication committee, data management team, and other individuals or groups overseeing the trial, if applicable (see Item 21a for data monitoring committee)
**Introduction**		
√ Background and rationale	6a	Description of research question and justification for undertaking the trial, including summary of relevant studies (published and unpublished) examining benefits and harms for each intervention
	6b	Explanation for choice of comparators
√ Objectives	7	Specific objectives or hypotheses

For peer review only - http://bmjopen.bmj.com/site/about/guidelines.xhtml

√ Trial design	8	Description of trial design including type of trial (eg, parallel group, crossover, factorial, single group), allocation ratio, and framework (eg, superiority, equivalence, noninferiority, exploratory)

Comment [UoN1]: We have chosen to put this in the methods section, if you prefer we will move it to the introduction, but we feel it flows better as the start of the method

Methods: Participants, interventions, and outcomes

√ Study setting	9	Description of study settings (eg, community clinic, academic hospital) and list of countries where data will be collected. Reference to where list of study sites can be obtained
√ Eligibility criteria	10	Inclusion and exclusion criteria for participants. If applicable, eligibility criteria for study centres and individuals who will perform the interventions (eg, surgeons, psychotherapists)
√ Interventions	11a	Interventions for each group with sufficient detail to allow replication, including how and when they will be administered
	11b	Criteria for discontinuing or modifying allocated interventions for a given trial participant (eg, drug dose change in response to harms, participant request, or improving/worsening disease)
	11c	Strategies to improve adherence to intervention protocols, and any procedures for monitoring adherence (eg, drug tablet return, laboratory tests)
	11d	Relevant concomitant care and interventions that are permitted or prohibited during the trial
√ Outcomes	12	Primary, secondary, and other outcomes, including the specific measurement variable (eg, systolic blood pressure), analysis metric (eg, change from baseline, final value, time to event), method of aggregation (eg, median, proportion), and time point for each outcome. Explanation of the clinical relevance of chosen efficacy and harm outcomes is strongly recommended
√ Participant timeline	13	Time schedule of enrolment, interventions (including any run-ins and washouts), assessments, and visits for participants. A schematic diagram is highly recommended (see Figure)
√ Sample size	14	Estimated number of participants needed to achieve study objectives and how it was determined, including clinical and statistical assumptions supporting any sample size calculations
√ Recruitment	15	Strategies for achieving adequate participant enrolment to reach target sample size

Methods: Assignment of interventions (for controlled trials)

Allocation:

√ Sequence generation	16a	Method of generating the allocation sequence (eg, computer-generated random numbers), and list of any factors for stratification. To reduce predictability of a random sequence, details of any planned restriction (eg, blocking) should be provided in a separate document that is unavailable to those who enrol participants or assign interventions
√ Allocation concealment mechanism	16b	Mechanism of implementing the allocation sequence (eg, central telephone; sequentially numbered, opaque, sealed envelopes), describing any steps to conceal the sequence until interventions are assigned
√Implementation	16c	Who will generate the allocation sequence, who will enrol participants, and who will assign participants to interventions
√ Blinding (masking)	17a	Who will be blinded after assignment to interventions (eg, trial participants, care providers, outcome assessors, data analysts), and how
	17b	If blinded, circumstances under which unblinding is permissible, and procedure for revealing a participant's allocated intervention during the trial
**Methods: Data collection, management, and analysis**		
√ Data collection methods	18a	Plans for assessment and collection of outcome, baseline, and other trial data, including any related processes to promote data quality (eg, duplicate measurements, training of assessors) and a description of study instruments (eg, questionnaires, laboratory tests) along with their reliability and validity, if known. Reference to where data collection forms can be found, if not in the protocol
	18b	Plans to promote participant retention and complete follow-up, including list of any outcome data to be collected for participants who discontinue or deviate from intervention protocols
√ Data management	19	Plans for data entry, coding, security, and storage, including any related processes to promote data quality (eg, double data entry; range checks for data values). Reference to where details of data management procedures can be found, if not in the protocol
√ Statistical methods	20a	Statistical methods for analysing primary and secondary outcomes. Reference to where other details of the statistical analysis plan can be found, if not in the protocol
	20b	Methods for any additional analyses (eg, subgroup and adjusted analyses)
	20c	Definition of analysis population relating to protocol non-adherence (eg, as randomised analysis), and any statistical methods to handle missing data (eg, multiple imputation)

Methods: Monitoring

√ Data monitoring	21a	Composition of data monitoring committee (DMC); summary of its role and reporting structure; statement of whether it is independent from the sponsor and competing interests; and reference to where further details about its charter can be found, if not in the protocol. Alternatively, an explanation of why a DMC is not needed
	21b	Description of any interim analyses and stopping guidelines, including who will have access to these interim results and make the final decision to terminate the trial
√ Harms	22	Plans for collecting, assessing, reporting, and managing solicited and spontaneously reported adverse events and other unintended effects of trial interventions or trial conduct
√ Auditing	23	Frequency and procedures for auditing trial conduct, if any, and whether the process will be independent from investigators and the sponsor

Ethics and dissemination

√ Research ethics approval	24	Plans for seeking research ethics committee/institutional review board (REC/IRB) approval
√ Protocol amendments	25	Plans for communicating important protocol modifications (eg, changes to eligibility criteria, outcomes, analyses) to relevant parties (eg, investigators, REC/IRBs, trial participants, trial registries, journals, regulators)
√ Consent or assent	26a	Who will obtain informed consent or assent from potential trial participants or authorised surrogates, and how (see Item 32)
	26b	Additional consent provisions for collection and use of participant data and biological specimens in ancillary studies, if applicable
√ Confidentiality	27	How personal information about potential and enrolled participants will be collected, shared, and maintained in order to protect confidentiality before, during, and after the trial
√ Declaration of interests	28	Financial and other competing interests for principal investigators for the overall trial and each study site
√ Access to data	29	Statement of who will have access to the final trial dataset, and disclosure of contractual agreements that limit such access for investigators
√ Ancillary and post-trial care	30	Provisions, if any, for ancillary and post-trial care, and for compensation to those who suffer harm from trial participation

For peer review only - http://bmjopen.bmj.com/site/about/guidelines.xhtml

√Dissemination policy	31a	Plans for investigators and sponsor to communicate trial results to participants, healthcare professionals, the public, and other relevant groups (eg, via publication, reporting in results databases, or other data sharing arrangements), including any publication restrictions
	31b	Authorship eligibility guidelines and any intended use of professional writers
	31c	Plans, if any, for granting public access to the full protocol, participant-level dataset, and statistical code
**Appendices**		
√ Informed consent materials	32	Model consent form and other related documentation given to participants and authorised surrogates
X Biological specimens	33	Plans for collection, laboratory evaluation, and storage of biological specimens for genetic or molecular analysis in the current trial and for future use in ancillary studies, if applicable

Comment [UoN2]: Not relevant

*It is strongly recommended that this checklist be read in conjunction with the SPIRIT 2013 Explanation & Elaboration for important clarification on the items. Amendments to the protocol should be tracked and dated. The SPIRIT checklist is copyrighted by the SPIRIT Group under the Creative Commons "Attribution-NonCommercial-NoDerivs 3.0 Unported" license.

For peer review only - http://bmjopen.bmj.com/site/about/guidelines.xhtml

[LOCAL TRUST LOGO]

Appendix A - CONSENT FORM FOR PARENTS
(Final version 1.2: 05.06.2014)

Title of Study: Assessing QbTest Utility in ADHD: A Randomised Controlled Trial

REC ref: ***14/WM/0166***

Name of Researcher:

Name of Participant (Parent):

Name of Participant (Child):

Please INITIAL box

1. I confirm that I have read and understand the information sheet version number 1.2 dated 05.06.2014 for the above study and have had the opportunity to ask questions. ☐

2. I understand that mine and my child's participation is voluntary and that we are free to withdraw at any time, without giving any reason, and without their medical care or legal rights being affected. I understand that should we withdraw then the information collected so far cannot be erased and that this information may still be used in the project analysis. ☐

3. I understand that relevant sections of my child's medical notes and data collected in the study may be looked at by authorised individuals from the University of Nottingham, the research group and regulatory authorities where it is relevant to our taking part in this study. I give permission for these individuals to have access to these records and to collect, store, analyse and publish information obtained from mine and my child's participation in this study. I understand that mine and my child's personal details will be kept confidential. ☐

4. I agree for my child's teacher and head teacher to be contacted and be asked to complete questionnaires about my child's behaviour at school. ☐

5. I understand if I/my child take part in any research interviews, they will be recorded and that anonymous direct quotes from the focus group may be used in the study reports. ☐

6. I agree for me and my child (named above) to take part in the above study. ☐

Name of Parent	Date	Signature

(OPTIONAL) Section for children to give assent

I agree to take part in this study

Name of Child (for assent)	Date	Signature

For peer review only

For peer review only - http://bmjopen.bmj.com/site/about/guidelines.xhtml

[LOCAL TRUST LOGO]

To be completed by researcher/investigator taking consent:

Researcher consent	Date	Signature

For peer review only - http://bmjopen.bmj.com/site/about/guidelines.xhtml

[LOCAL TRUST LOGO]

Appendix B - CONSENT FORM FOR YOUNG PEOPLE
(Final version 1.2: 05.06.2014)

Title of Study: Assessing QbTest Utility in ADHD: A Randomised Controlled Trial

REC ref: ***14/WM/0166***

Name of Researcher:

Name of Participant (young person):

Name of Participant (parent):

Please <u>INITIAL</u> box

1. I confirm that I have read and understand the information sheet version number 1.2 dated 05.06.2014 for the above study and have had the opportunity to ask questions. ☐

2. I understand that my participation is voluntary and that I am free to withdraw at any time, without giving any reason, and without their medical care or legal rights being affected. I understand that should they withdraw then the information collected so far cannot be erased and that this information may still be used in the project analysis. ☐

3. I understand that relevant sections of my medical notes and data collected in the study may be looked at by authorised individuals from the University of Nottingham, the research group and regulatory authorities where it is relevant to our taking part in this study. I give permission for these individuals to have access to these records and to collect, store, analyse and publish information obtained from my child's participation in this study. I understand that my child's personal details will be kept confidential. ☐

4. I agree for my teacher and head teacher to be contacted and be asked to complete questionnaires about my behaviour at school. ☐

5. I understand if I/ take part in any research interviews, they will be recorded and that anonymous direct quotes from the focus group may be used in the study reports. ☐

6. I agree to take part in the above study. ☐

Name of young person	Date	Signature

To be completed by researcher/investigator taking consent:

Name of researcher	Date	Signature

Assessing QbTest Utility in ADHD: A Randomised Controlled Trial V1.2 05.06.2014

For peer review only

Figure 1: Study Flowchart

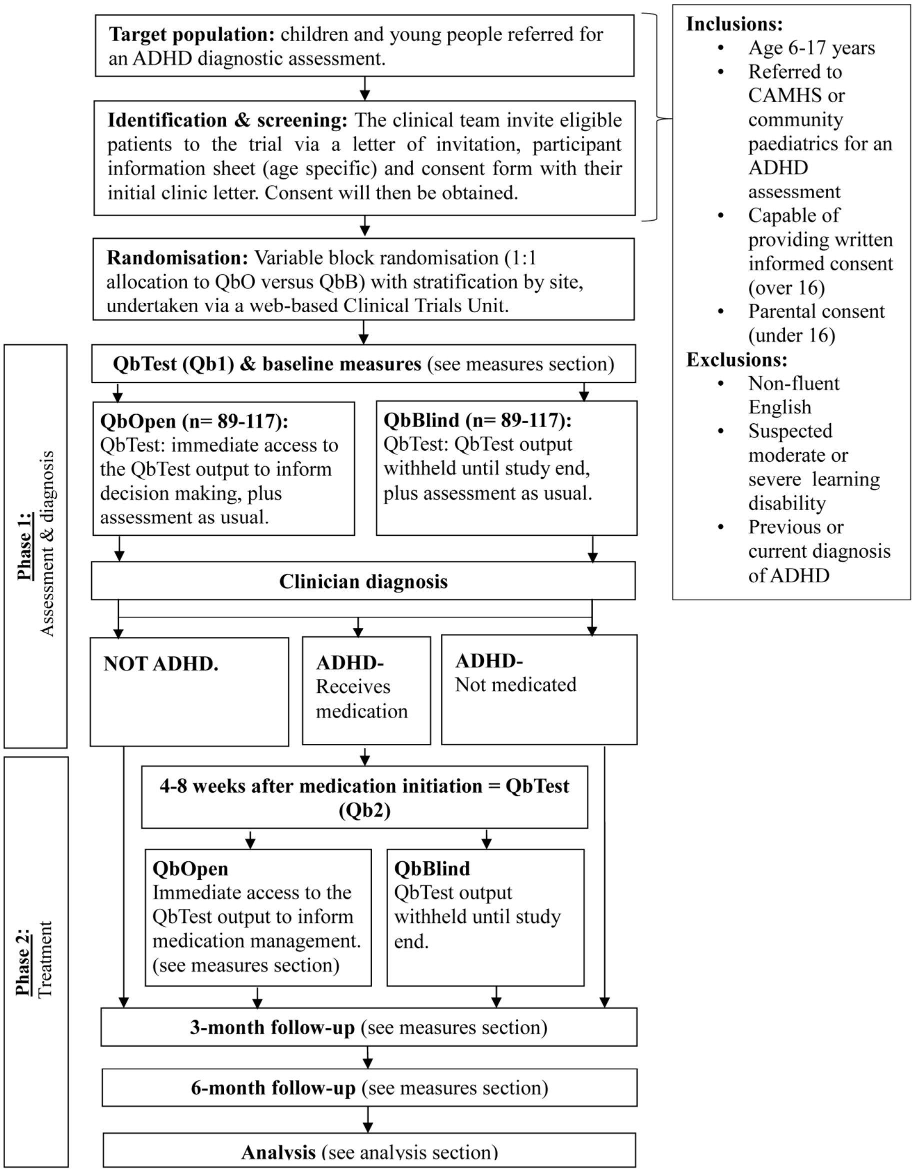

226x294mm (300 x 300 DPI)
