# Supplementary material for: Protocol investigating the clinical utility of an objective measure of activity and attention (QbTest) on diagnostic and treatment decision-making in children and young people with ADHD—‘Assessing QbTest Utility in ADHD’ (AQUA): a randomised controlled trial
Source: BMJ Open. 2014 Dec 1;4(12):e006838. doi: 10.1136/bmjopen-2014-006838 (PMC4256543; doi:10.1136/bmjopen-2014-006838)
Supplement: Reviewer comments [file bmjopen-2014-006838.reviewer_comments.pdf]

PEER REVIEW HISTORY

BMJ Open publishes all reviews undertaken for accepted manuscripts. Reviewers are asked to complete a checklist review form (**http://bmjopen.bmj.com/site/about/resources/checklist.pdf**) and are provided with free text boxes to elaborate on their assessment. These free text comments are reproduced below.

ARTICLE DETAILS

**TITLE (PROVISIONAL)**	Protocol investigating the clinical utility of an objective measure of activity and attention (QbTest) on diagnostic and treatment decision-making in children and young people with ADHD: 'Assessing QbTest Utility in ADHD' (AQUA) a randomised controlled trial
**AUTHORS**	Hall, Charlotte; Walker, Gemma; valentine, althea; Guo, Boliang; Kaylor-Hughes, Catherine; james, marilyn; daley, david; Sayal, Kapil; Hollis, Chris

This article was not externally reviewed at BMJ Open. Protocols that have been independently assessed prior to submission to BMJ Open are usually fast-tracked to publication on the grounds that further substantial changes will not be possible. This independent assessment will usually be external, independent review for both a substantial grant award from a non-commercial or government funder and ethics approval.
